# Bushveld superplume drove Proterozoic magmatism and metallogenesis in Australia

**DOI:** 10.1038/s41598-020-76800-0

**Published:** 2020-11-12

**Authors:** Marco L. Fiorentini, Craig O’Neill, Andrea Giuliani, Eunjoo Choi, Roland Maas, Franco Pirajno, Stephen Foley

**Affiliations:** 1 School of Earth Sciences, University of Western Australia, 35 Stirling Hwy, Crawley, WA 6009 Australia; 2 Department of Earth and Environmental Sciences, Macquarie University, Sydney, NSW 2109 Australia; 3 Department of Earth Sciences, Institute of Geochemistry and Petrology, ETH Zurich, Clausiusstrasse 25, 8092 Zurich, Switzerland; 4 School of Earth Sciences, University of Melbourne, Parkville, VIC 3010 Australia

**Keywords:** Geochemistry, Geodynamics, Geology, Petrology

## Abstract

Large-scale mantle convective processes are commonly reflected in the emplacement of Large Igneous Provinces (LIPs). These are high-volume, short-duration magmatic events consisting mainly of extensive flood basalts and their associated plumbing systems. One of the most voluminous LIPs in the geological record is the ~ 2.06 billion-year-old Bushveld Igneous Complex of South Africa (BIC), one of the most mineralised magmatic complexes on Earth. Surprisingly, the known geographic envelope of magmatism related to the BIC is limited to a series of satellite intrusions in southern Africa and has not been traced further afield. This appears inconsistent with the inferred large size of the BIC event. Here, we present new radiometric ages for alkaline magmatism in the Archean Yilgarn Craton (Western Australia), which overlap the emplacement age of the BIC and indicate a much more extensive geographic footprint of the BIC magmatic event. To assess plume involvement at this distance, we present numerical simulations of mantle plume impingement at the base of the lithosphere, and constrain a relationship between the radial extent of volcanism versus time, excess temperature and plume size. These simulations suggest that the thermal influence of large plume events could extend for thousands of km within a few million years, and produce widespread alkaline magmatism, crustal extension potentially leading to continental break-up, and large ore deposits in distal sectors. Our results imply that superplumes may produce very extensive and diverse magmatic and metallogenic provinces, which may now be preserved in widely-dispersed continental blocks.

## Introduction

The convective processes responsible for the dispersion of heat from the Earth’s core to its outer surface are reflected in the formation of mantle plumes^1,2^, which can result in the emplacement of enormous amounts of magma into the crust over very short time spans, generally less than 5 Ma^3,4^. During the evolution of our planet from the Archean to the present, the size and magnitude of mantle plumes has varied greatly, reflecting both secular cooling and the cyclic assembly and breakup of supercontinents^5,6^. Geological evidence shows that deep mantle plume activity and associated high-volume magmatism have strong impacts on the continental crust and the Earth’s surface, e.g. through their role in continental break-up, formation of large ore deposits and as drivers of dramatic environmental change^7,8^.

The Bushveld Igneous Complex (BIC) of South Africa, which hosts the largest known reserves of platinum group elements (PGEs), vanadium and chromium, is thought to reflect the arrival of a large mantle plume under the Kaapvaal Craton at ~ 2.06 Ga^9^. This craton had already formed a deep, buoyant and refractory lithospheric root by ~ 3 Ga^10^. The BIC is best known for preserving an extremely thick (> 7 km) sequence of mafic sills and ultramafic cumulates injected sequentially into the Transvaal Supergroup^11^. In addition, the BIC comprises large volumes of felsic volcanic and granitic rocks (> 90,000 km^3^ of granite/granophyre^12^). While details of their formation are still debated, it is generally assumed that the layered mafic/ultramafic rocks represent an upward-aggrading pile of crystals deposited on the floor of a vast, long-lived and repeatedly replenished magma chamber^13^. Whereas the upward-aggrading model has been challenged^9^, it is widely accepted that the entire magmatic stratigraphy of the BIC was formed over a time span of less than 5 Ma^14^, and most likely within only 0.1–0.2 Ma, at ~ 2055 Ma^15,16^.

**Figure 1:**
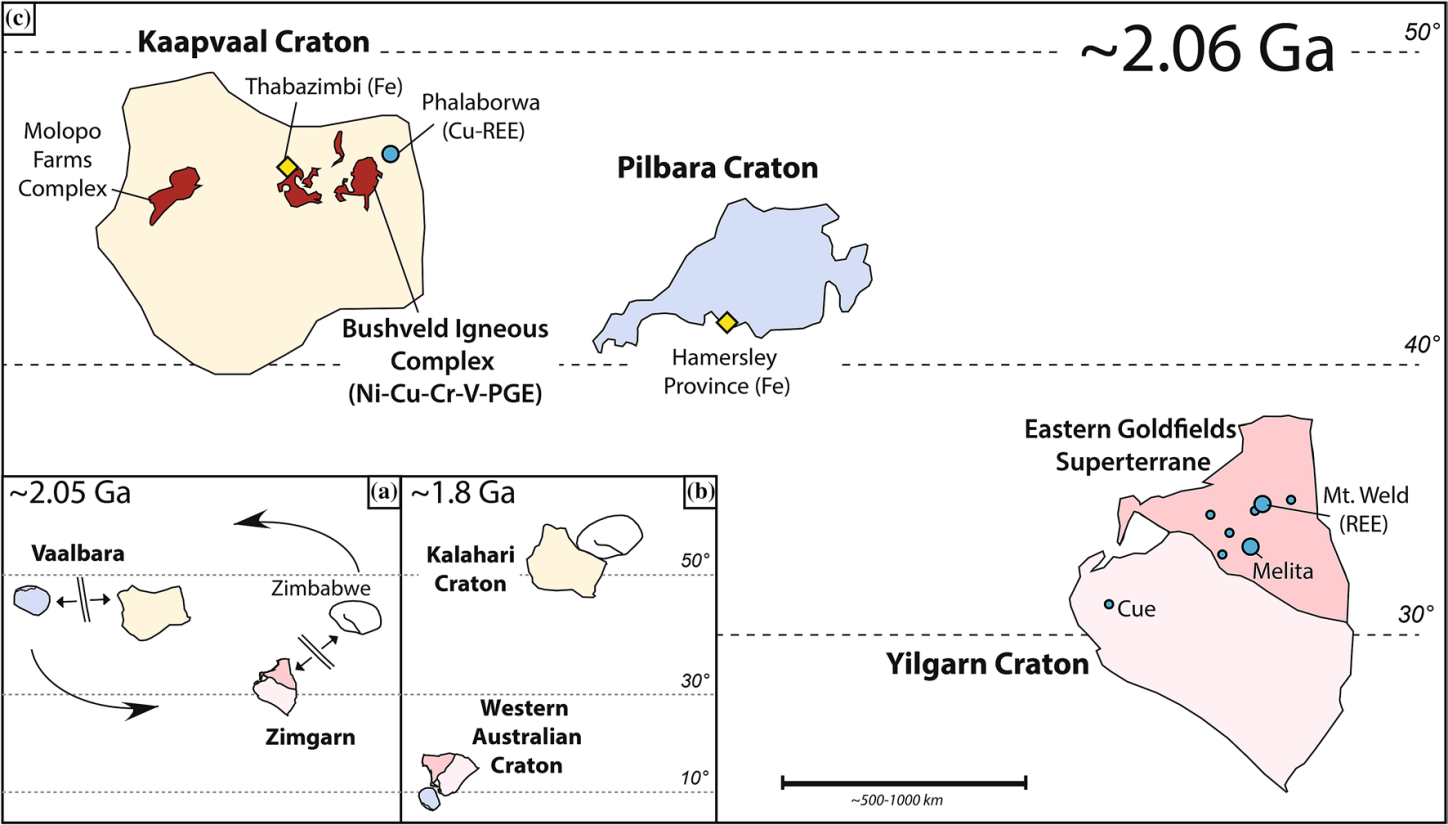
(**A**) Reconstruction of the Vaalbara and Zimgarn cratons in the early stages of break-up, at ~ 2.06 Ga. (**B**) Kalahari and West Australian cratons after final assembly, as early as ~ 1.95 Ga. (**C**) Inferred relative position of Yilgarn, Kaapvaal, and Pilbara cratons at ~ 2.06 Ga (relative distance between BIC and EGS is between 3000 and 4000 km). Modified after Smirnov et al.^37^. This figure was created in Adobe Illustrator (www.adobe.com).

The size and short emplacement history of the BIC are consistent with formation above a large mantle plume, possibly a superplume, characterised by a radius of surface extent of > 1000 km^17^. In addition to the large volume of melts at their centre, the impingement of superplumes at the base of the lithosphere is expected to generate small-scale magmatism distal from the plume centre^18,19^. However, syn-BIC magmatism appears to be limited to the Molopo Farms Complex and to a few satellite intrusions within a radius of < 500 km from the inferred plume centre (Fig. 1), assumed to be located underneath the BIC^8^. This apparent lack of a spatially extensive magmatic footprint appropriate to the inferred large size of the BIC event is surprising and possibly related to dispersal of part of the syn-Bushveld magmatic record through plate tectonicic activity.

Here, we present new emplacement ages for Paleoproterozoic alkaline magmatic complexes in the Yilgarn Craton of Western Australia, showing their overlap with the formation of the BIC. Arguing a genetic link between these units is challenging as the reconstructed positions of the Kaapvaal and Yilgarn cratons are uncertain at this time; their distance is also not clear, but has been suggested to be > 2000–3000 km, far outside the radial influence of any Phanerozoic plume. No interposed volcanics have been preserved, as the intervening oceanic crust has been destroyed. Furthermore, the spreading plume would have had to navigate the sublithospheric architecture of oceanic crust and cratonic roots^20^, complicating its passage. To assess the plausibility of a Bushveld superplume origin for the Yilgarn alkaline magmas, we have developed numerical simulations of mantle plume impingement at the base of a heterogenous lithosphere. We have used geochronological constraints and excess temperature estimates from magma compositions to constrain a relationship between the radial extent of volcanism versus time, and plume size. These results suggest that the Yilgarn alkaline province may be a distal expression of the Bushveld superplume, which was separated from its former location closer to the Kaapvaal Craton by subsequent terrane dispersal.

### Geological settings and existing geochronological constraints

Kimberlites, ultramafic lamprophyres and carbonatites in the heavily-mineralised Eastern Goldfields Superterrane (EGS) of the Archean Yilgarn Craton occur over an area of 45,000 km^2^. Largely unpublished radiometric dating suggested a ~ 2 Ga age for the province (see details in Supplementary Material). This was confirmed by Re-Os isotope data for whole rocks and oxide minerals (ilmenite, spinel), which indicate an age of 2025 ± 10 Ma^21^. Homogenous initial Os (γ_Os_ =  + 5.1 ± 3.1) and Nd (ε_Nd_ =  + 0.1 to + 1.6) isotope ratios suggested similar mantle sources over the large area covered by these alkaline rocks^21^. Although Re-Os dating of kimberlites is problematic—Re and Os are known to be hosted in both magmatic minerals and possibly unequilibrated xenocrysts^22,23^—the ~ 2.02 Ga apparent age for the EGS alkalic complexes provided encouragement to further radiometric dating, with the aim of searching for syn-Bushveld magmatic activity in the Yilgarn Craton.

### Methodology

Phlogopite Rb–Sr and apatite U–Pb ages were acquired for two members of the EGS Proterozoic alkaline province (Fig. 1), the Mt. Weld carbonatite and Melita ultramafic lamprophyre. Both minerals were characterised by electron microscopy, electron microprobe and laser-ablation ICP-MS at the University of Western Australia, Perth. Rb–Sr and U–Pb ages were acquired at the University of Melbourne, using isotope dilution MC-ICP-MS and laser-ablation ICP-MS, respectively. Details on the geology of these intrusions, the studied samples and the analytical methods are provided in the Supplementary Material.

The numerical simulations were based on the community code Aspect^24^, which was used to simulate the rise of initial spherical anomalies from the core-mantle boundary (CMB), and the interaction of plumes with surface lithospheric structures such as cratonic roots. We assume a thermal field appropriate for the Proterozoic (see Supplementary Material), and impose strong, buoyant cratonic blocks at the surface. The temperatures at the CMB are perturbed to invigorate plume formation. We then solve general compressible convection equations for mass, momentum and energy conservation. The energy equation incorporates decaying radioactive heating sources as well as shear and adiabatic heating. Material properties such as density, heat capacity, thermal expansivity and compressibility are calculated using lookup tables, and other key parameters are shown in the Supplementary Material (Table S6). We use a composite viscosity based on four deformation mechanisms: diffusion creep, dislocation creep, Peierls creep (which take an Arrhenius form) and yielding (which follows a Byerlee-style law^25^). The rheological parameters used in our models are shown in the Supplementary Material (Table S7).

## Results

**Figure 2:**
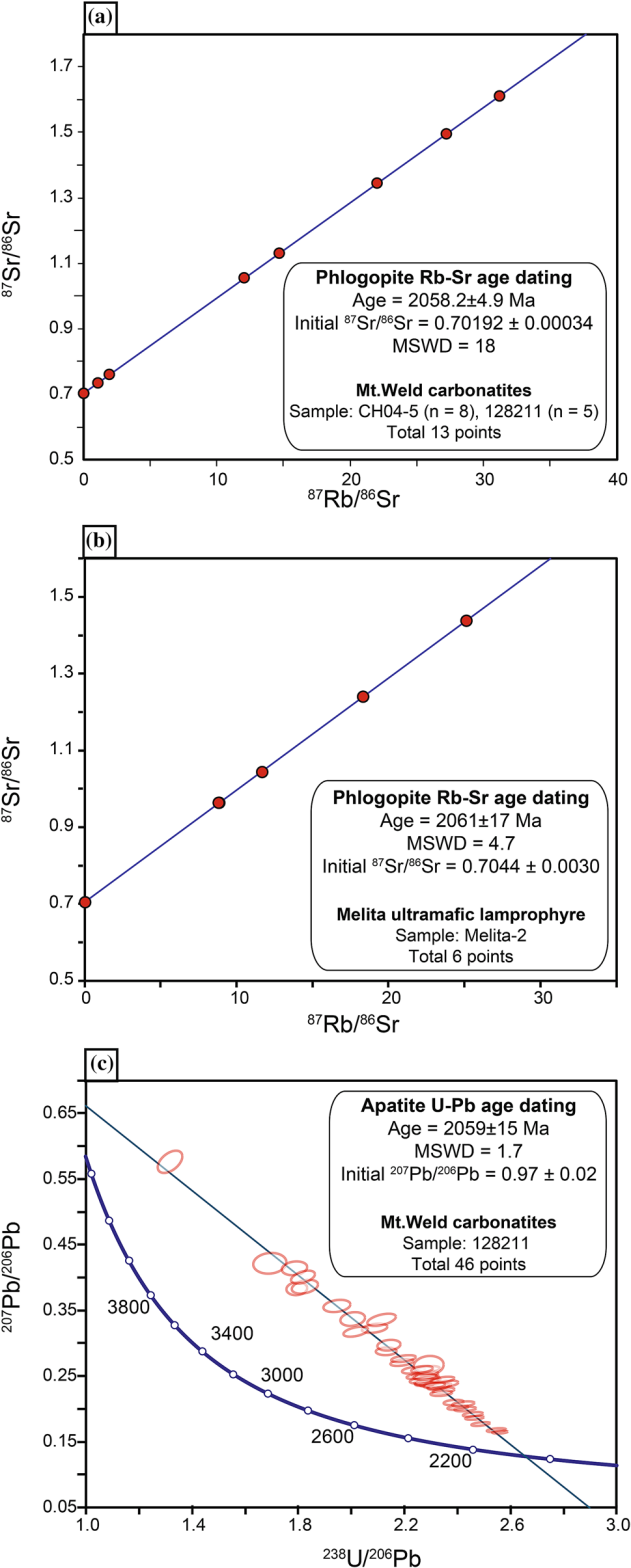
Rb–Sr (phlogopite) and U–Pb (apatite) geochronological data for (**A**,**C**) the Mt. Weld carbonatite and (**B**) Melita ultramafic lamprophyre. The isochron was generated using IsoplotR^61^ and the figure was created in Adobe Illustrator (www.adobe.com). Complete datasets are provided in Supplementary Material.

Rb–Sr dating of magmatic phlogopite from the Mt. Weld carbonatite yields an age of 2058.2 ± 4.9 Ma (95% CL, n = 13, MSWD = 1.9, initial ^87^Sr/^86^Sr = 0.70192 ± 0.00034; Fig. 2 and Table S3 in the Supplementary Material). This result is supported by a 2059 ± 15 Ma U–Pb age for phenocrystic and groundmass apatite from a porphyritic carbonatite from the Mt. Weld complex (95% CL, n = 46, MSWD = 1.7, initial ^207^Pb/^206^Pb = 0.97 ± 0.02; Fig. 2 and Table S5 in the Supplementary Material). Rb–Sr dating of phlogopite phenocrysts from the Melita ultramafic lamprophyre yields a 6-point isochron corresponding to an age of 2061 ± 17 Ma (95% CL, MSWD = 4.7, initial ^87^Sr/^86^Sr = 0.7044 ± 0.0030; Fig. 2 and Table S3 in the Supplementary Material). The emplacement age of the Mt. Weld carbonatite^26^ and Melita ultramafic lamprophyre are indistinguishable from each other and from the timing of Bushveld LIP formation. Accordingly, they may have been emplaced coevally with the impingement of the ~ 2.06 Ga superplume underneath the Kaapvaal Craton.

To assess the plausibility of the proposed influence of the Bushveld superplume in the distal alkaline province in the Yilgarn Craton, we have simulated the interaction of variably-sized plumes underneath cratonic lithosphere. Two principal constraints are available for these simulations. The first of these is a time interval of ~ 5 Ma for the timing of the magmatic events in the BIC and EGS provided by the radiometric ages. This implies a trade-off between the plume head size and the original distance between the BIC and the EGS alkaline province, and their respective host cratons. The second constraint is the excess temperature of the Bushveld superplume, which is related to the initial plume head volume flux and thus size of the plume^27^.

**Figure 3:**
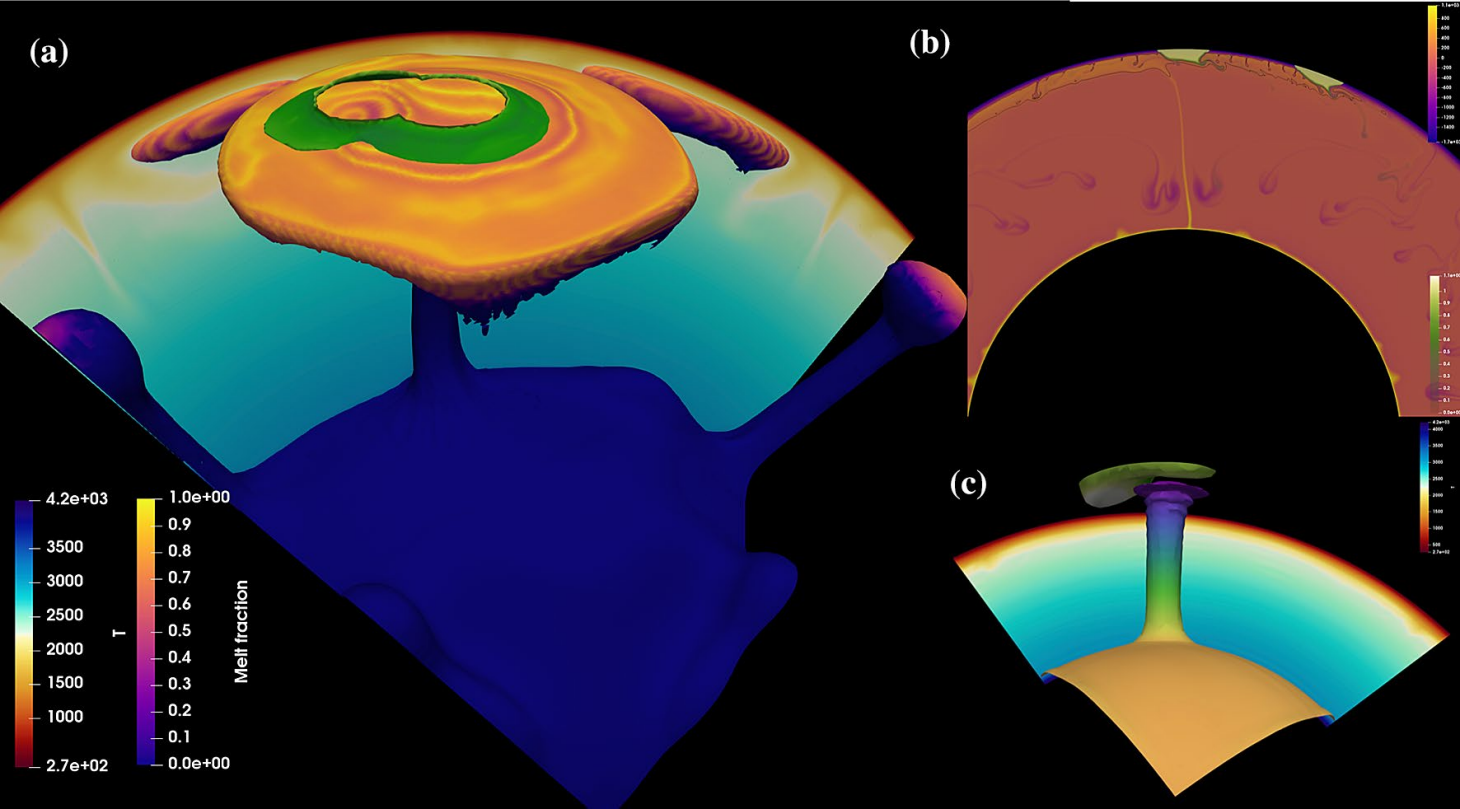
(**a**) Perspective view of the 3D simulation of the Bushveld plume impinging on the Kaapvaal–Pilbara craton, using the community code *Aspect.* Initial conditions were based on inferred continental configurations (Fig. 1) and are discussed in the Supplementary Material. The model examines a plume under the cratonic blocks, with an initial head radius of ~ 450 km in the lower mantle, which expands to > 3000 km radius at the lithosphere-asthenosphere boundary. The background slice shows the temperature colour-scheme, and the contoured plumes are coloured according to maximum melt fraction reached. The structure of the cratonic keels (green, note melting is occurring within the lithospheric root in this timestep) exerts control over the melting dynamics, as the plume advects around the keel structures. However, a plume of this size is capable of crossing multiple deep roots and generating low-fraction melt at large distances from its point of impact; (**b**) 2D simulation of the effect of a large plume (lower mantle radius 203.5 km) on distal magmatism. Colours represent temperature field, and compositionally distinct cratonic roots (green shades). 5% melt contour is shown as a black line; simulation shown after 12 Myr evolution; (**c**) Simulation with free surface boundary condition, demonstrating rift-related lithosphere flow and thinning of the imposed cratonic block in response to plume impingement on the lithosphere-asthenosphere boundary. Simulation performed and figure created with with geodynamics code Aspect 2.0 (https://aspect.geodynamics.org).

Excess temperature in BIC parental melts may be estimated from their MgO contents, which were modelled to have ranged from 29 to 19 wt% (and probably between 27 and 21 wt%^28^). This suggests primary melt temperatures of 130–330 °C above the ambient mantle temperature (~ 1250 °C^29^). Plume head diameter is a function of entrainment of mantle material during ascent and spreading at the base of the lithosphere (Fig. 3a), but also of the initial volume of material at the CMB. The latter may be modulated by variations in subduction history and slab dynamics at the CMB, the period of thermal boundary layer in-growth at the core and the presence of large low-shear-velocity provinces (LLSVPs^30^), all of which may have varied over Earth’s history.

The simulations carried out here show that relatively small plumes (< 200 km initial diameter at the CMB) either do not rise through the mantle without being mixed, or they do not cover lateral distances >  ~ 2000 km within 5 Ma after arriving at the lithospheric boundary. In contrast, our results show that a superplume with > 800 km initial diameter may rise and spread very rapidly. It would cover lateral distances of ~ 2500 km within < 1 Myr and have the capacity to generate melt fractions of ~ 5%, which are required for alkaline magmatism, at distances > 2500 km (Fig. 3b). It should be noted that these melts originate in the plume head itself. However, many alkaline magmas and carbonatites are suggested to originate in the subcontinental lithospheric mantle (SCLM), which is metasomatised by upwelling asthenospheric melts enriched by C, F, Cl and S^31^ and display domains of variable *f*O_2_. The metasomatised SCLM partially melts producing alkaline and carbonatitic magmas. Accordingly, we also explore lithospheric melting in the Supplementary Material. The key observation here is that it is the magnitude of the plume that dictates the extent of both lithospheric and asthenospheric melting, and thus our primary distance constraints are the same in either case.

**Figure 4:**
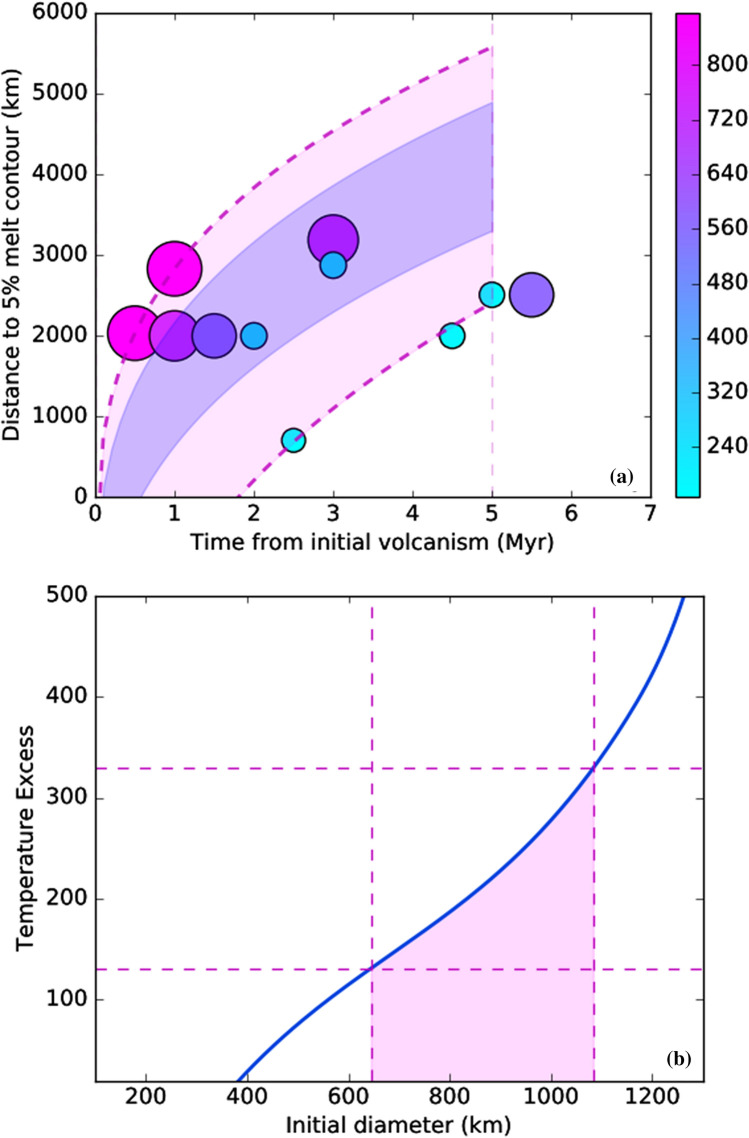
(**a**) Aggregation of numerical results for plume simulations (coloured circles), showing the time it takes for the plume to travel laterally from its sublithospheric point of impact, against the maximum distance to the 5% melt contour from the centre of the plume. The circles are results from individual simulations, and their scale and colour reflect their initial plume diameters (d) in the lower mantle (colour bar, in km). Large plumes (d > 800 km) move laterally very rapidly, whilst small plumes (d < 200 km) spread laterally much more slowly, and in some cases are not able to travel 2000 km within 5 million years. The scatter between runs is due to small-scale convection initiating in spreading plumes. Dashed magenta lines show interpolated trajectories based on buoyancy scalings, for d = 877 km (top) and 187 km (bottom). Blue shaded area shows highest likelihood range of extent for plumes between 650 and 800 km. Maximum range of plume influence within 5 million years (the geochronological constraint on the melts) is between 2200 and 5500 km; (**b**) The relationship between plume temperature excess, and its initial diameter, based on Eq. (1)^27^, and fitted to our modelled source volume fluxes. The permitted temperature excesses from the Bushveld parental magma compositions are 130–330 K, which limit the initial plume diameters to between 644 and 1083 km. This, combined with constraints on upper size of the plume from fluid dynamics theory, further constrains the extent of plume influence to within ~ 3000–4000 km (**a**). Simulation performed and figure created with with geodynamics code Aspect 2.0 (https://aspect.geodynamics.org).

In Fig. 4, we have plotted our numerical results for plume spreading under heterogenous on- and off-craton lithosphere. We have also used a scaling for the plume head buoyancy flux (related to the plume head thickness h), which scales with the velocity of the plume front, to constrain and extrapolate the lateral extent of plume heads within the new geochronological constraints presented in this study (5 Myr). This scaling assumes a constant volume plume head beneath the lithosphere (~ hr^2^, where r is the radius and h is its thickness), which spreads in a cylindrical fashion under its buoyancy (which itself scales with h). This geometrical configuration requires r ~ t^1/3^ (dashed lines in Fig. 4). For the range of initial plume sizes explored here (100–1000 km starting plumes to span the plausible natural range), we find that significant alkaline magmatism (tracked by the 5% melt contour) is generated at distances of up to 2200–5500 km from the plume centre, within the 5 Ma time constraint (Fig. 4a).

An additional constraint on the size of the plume head and its distal influence may be made using its excess temperature upon arrival under the lithosphere. We calculated the source volume flux of our initiating plumes (from the simulation flow fields within the incipient plumes) and then used the scaling relations of Griffith and Campbell^27^ (Eq. 1) to derive a relationship between the size of our starting plume and the temperature excess upon impingement at the base of the lithosphere.

1 $$ {\raise0.7ex\hbox{${\Delta T}$} \!\mathord{\left/ {\vphantom {{\Delta T} {\Delta T_{S} }}}\right.\kern-\nulldelimiterspace} \!\lower0.7ex\hbox{${\Delta T_{S} }$}} = \left( {{\raise0.7ex\hbox{${27}$} \!\mathord{\left/ {\vphantom {{27} {80}}}\right.\kern-\nulldelimiterspace} \!\lower0.7ex\hbox{${80}$}}} \right)\left( {{\raise0.7ex\hbox{$6$} \!\mathord{\left/ {\vphantom {6 \pi }}\right.\kern-\nulldelimiterspace} \!\lower0.7ex\hbox{$\pi $}}} \right)^{{2/3}} C^{{ - 2}} \left( {{\raise0.7ex\hbox{$Q$} \!\mathord{\left/ {\vphantom {Q \kappa }}\right.\kern-\nulldelimiterspace} \!\lower0.7ex\hbox{$\kappa $}}} \right)z^{{ - 1}}  $$

Griffiths and Campbell^27^ assumed that C ~ 2, $\Delta {T}_{S}$ is their initial plume temperature excess (400 K), κ is the thermal diffusivity (5.8 × 10^−6^), and z refers to the mantle depth (2890 km). The permissible temperature excesses based on the estimates of primary melt MgO from Wilson^28^ are consistent with initial plume diameters in the range 644–1083 km in the lower mantle. This is near the upper end of possible sizes of growing plumes, based on Rayleigh–Taylor stability considerations^32^. The head of a plume this size would pancake to radii of over ~ 2000 km immediately upon hitting the lithosphere, and spread further still under lateral buoyancy forces. This constrains the maximum distance between the plume centre (BIC) and its most distal lateral magmatic effects (EGS alkaline province) to 3000–5500 km, or, for most likely plume sizes (600–800 km), between around 3000–4000 km (Fig. 4a).

## Discussion

The new emplacement ages for Mt. Weld and Melita indicate that these intrusions, together with other widely-dispersed and isotopically similar alkaline complexes (kimberlites, ultramafic lamprophyres) in the EGS, represent an extensive alkaline province formed at ~ 2.06 Ga^21^. No evidence for magmatic or tectonic triggers of alkaline magmatism at ~ 2.06 Ga is preserved in the Yilgarn Craton^21^ or anywhere in the Australian continent^33^. Here, we explore the hypothesis that alkaline magmatism in the EGS may instead be genetically related to the Bushveld superplume. A remarkable (and coeval) example of such a relationship is provided by the link between the BIC and the Phalaborwa and Shiel carbonatites in the Kaapvaal Craton^19^.

A critical aspect of this hypothesis, the relative position of the Kaapvaal and Yilgarn cratons at ~ 2.06 Ga, is poorly constrained^34–36^. Some evidence suggests that prior to ~ 2.2 Ga, the Kaapvaal and Pilbara cratons formed the ‘Vaalbara Craton’, whereas the Yilgarn and Zimbabwe cratons formed a separate continental block, the ‘Zimgarn Craton’^37^. Break-up of Vaalbara may have occurred as early as ~ 2.2 Ga, with widespread magmatism in both the Kaapvaal^38^ and Pilbara cratons^39^. In contrast, if break-up of Vaalbara was coeval with the BIC event^37^, the Kaapvaal and Yilgarn cratons may have been relatively close to each other at ~ 2.06 Ga, as Vaalbara and Zimgarn “traded partners” prior to break-up and subsequent amalgamation into the Kalahari and West Australian cratons perhaps as early as ~ 1.95 Ga^35,37,40^. However, robust estimates of the distance between Vaalbara and Zimgarn at ~ 2.06 Ga are not available, mainly due to the paucity of reliable paleomagnetic poles. Our new data may shed light on this knowledge gap.

The numerical models presented here predict that partial melting within the lateral extent of the BIC plume depends on the size of the plume, defined as the plume’s diameter in the lower mantle. Fluid dynamics would limit effective plume diameters in the lower mantle to between 100 km (below which plume heads are entrained) and ~ 1000 km (the upper range of Rayleigh–Taylor stability^41^). A ~ 200 km diameter plume is able to rise through the mantle and spread at the base of the lithosphere, but typically it will not spread rapidly, due to the small lateral buoyancy force, and it will not spread far: only a small number of our model simulation runs for plumes this size spread 2000 km within the geochronological constraint of 5 Ma. By contrast, the largest quasi-stable examples (the largest plumes at the limit of stability for extreme lower-mantle viscosities) in our simulations have lower mantle diameters around 800 km. Such extreme plumes rapidly grow due to entrainment during ascent, and rapidly spread laterally at the lithospheric boundary, transiting up to >  ~ 5500 km within 5 Ma (Fig. 4a).

Inferred excess temperatures of BIC parental magmas, based on magma MgO contents of Wilson^28^, limit the maximum size of the BIC plume to ~ 650–1000 km, and most likely 650–800 km^27^. This range in plume sizes results in estimates for the maximum extent of plume-related melting between 3000 and 4000 km after 5 Ma. In our model, this would provide an upper limit to the distance between the Kaapvaal and Yilgarn cratons at ~ 2.06 Ga. Low-degree melts such as kimberlites and ultramafic lamprophyres could be generated anywhere within this large thermal anomaly even though peripheral locations are more likely^19^. For example, low-degree melting could slightly precede the major phase of melting and be associated with the initial thermal anomaly associated with plume upwelling but before plume impingement on the overlying lithosphere. The minimum distance between the Kaapvaal and Yilgarn is not tightly constrained here: it is effectively provided by the size of the two cratons at this time.

However, it should be considered that one constraint on the minimum distance between the Kaapvaal and Yilgarn cratons at ~ 2.06 Ga is the exclusively alkaline-ultramafic composition of the EGS Paleoproterozoic magmatic province, a ca. 500 × 150 km linear belt^21^. In fact, the apparent absence of subalkaline mafic magmatism at this time may indicate a minimum distance between the two cratons. Basalt petrogenesis involves higher degrees of mantle melting than those required for alkaline magmatism, limiting plume-related basaltic magmatism to maximum distances of ~ 2000 km from the superplume centre beneath the BIC in Vaalbara^8^. The petrogenesis of ultramafic lamprophyres involves low-degree (f ≤ 0.05) partial melting of the mantle^42^, which could occur anywhere between plume centre and the periphery of the plume’s thermal influence.

As long as the paleo-distance between the relevant locations in the Kaapvaal and Yilgarn cratons did not exceed 3000–4000 km, the Bushveld superplume would be able to cover the lateral distance effectively even in the presence of sublithospheric topography^20^, advecting past the edges of Vaalbara and possible intervening oceanic domains, and eventually reach the EGS (Figs. 1 and 3b). Generation of the plume-related magmas involved in the EGS alkaline province may occur as melting of peridotite in the plume head itself^43^ or in the lower lithosphere^42^. The latter would be volatile-driven melting of easily fusible materials, but enhanced by plume head energy (Supplementary Material). In both cases, the lateral extent of melting is largely constrained by the spatial bounds of the plume material. In the specific scenario discussed here, it is inferred that the alkaline rocks in the EGS are associated with low-degree partial melting of the convective uppermost asthenosphere or the base of the cratonic lithosphere^21,44–46^.

The syn-BIC alkaline province in the EGS does not appear to be matched by similarly prominent syn-BIC magmatism in other, older, parts of the Yilgarn Craton. It is possible that further Paleoproterozoic alkaline (or even subalkaline) magmatic complexes elsewhere in the Yilgarn Craton were eroded, or have not yet been discovered due to poor exposure. For example, remnants of a 1.89 Ga mafic magmatic event in the Yilgarn Craton were only discovered recently^36^. However, given the broadly homogeneous type, nature and thickness of the regolith cover across the craton, including the EGS, we suggest that other factors, such as pre-existing lithospheric architecture, play a first-order role in focussing the crustal expression of plume-related magmatism.

It is known that mantle-derived carbonate-rich magmas are preferentially emplaced at craton margins^42^ or along lithospheric sutures^47^ where they can exploit trans-lithospheric corridors to ascend to upper crustal levels. Due to its thinner and more juvenile lithospheric architecture compared to adjacent areas of the Yilgarn and Pilbara cratons^48–50^, the EGS was probably a favourable locus for the generation and emplacement of the alkaline magmas associated with the distal Bushveld superplume. Limited partial melting of enriched domains could have occurred in the deep lithosphere of the thicker western portion of the Yilgarn Craton, but be scarcely represented at surface. The only known hypabyssal calc-alkaline lamprophyres in that part of the craton, at Cue in the northwestern Yilgarn Craton (Fig. 1), were emplaced at ~ 2.06 Ga^50^ and could represent an additional distal expression of the BIC superplume in Zimgarn.

The arrival of the inferred Bushveld superplume at the base of Vaalbara may have triggered the break-up of this long-lived, thick and buoyant lithospheric block^37^ (Fig. 3c). While it is difficult to estimate the far-field plate forces or plate motions at the time of emplacement of the plume, a series of free-surface models demonstrate that a plume of > 400 km lower mantle diameter would drive significant lateral motion, lithosphere root erosion, local thinning/rifting, and ultimately craton break-up (Fig. 3c). An even larger plume (650–800 km as inferred here for the BIC superplume), would therefore be expected to have profound tectonic effects, perhaps including the break-up of Vaalbara to form the separate Kaapvaal and Pilbara cratons. Possible evidence of lithosphere-scale processes may be preserved in the form of extensional sedimentary basins, such as the Horseshoe Basin in the Pilbara Craton. This intracontinental rift basin formed between ~ 2.15 and 2.01 Ga^51^ and preserves up to 360 m of basal fluvial and shallow-marine siliciclastic sandstones, ∼2.7 km of flood basalt and ≥ 325 m of platform carbonates and associated volcaniclastic and siliciclastic sediments.

The thermal anomaly associated with the ~ 2.06 Ga Bushveld superplume may also have driven geographically extensive hydrothermal activity, perhaps associated with deep-crustal magmatism. By analogy with the Thabazimbi iron deposits in the Kaapvaal Craton (Fig. 1), for which a genetic link with the BIC had been established, Taylor et al.^52^ suggested that high-grade iron mineralisation in the Hamersley Province (southern margin of Pilbara Craton, Fig. 1) was generated coevally with the deposits at Thabazimbi, shortly before 2.0 Ga. Extensive syn-BIC hydrothermal activity associated with incipient rifting and associated intrusive activity shortly after 2.06 Ga may thus be responsible for an important hypogene upgrading of the 2770–2410 Ma sedimentary banded iron formations of the Hamersley Province (see also Krapež et al.^51^). The thick lithosphere of the Pilbara Craton may have prevented eruption of significant volumes of mafic magmas synchronously with this activity^49^. However, our models suggest that after plume-induced thinning of the cratonic roots, ongoing smaller-scale convection in the upper asthenosphere may have driven secondary volcanism. The 2031 ± 6 Ma Cheela Springs Basalt in the Pilbara Craton may represent a delayed magmatic expression of the Bushveld superplume^51^.

A genetic link of high-grade iron ore along the southern margin of the Pilbara Craton with magmatic activity triggered by the Bushveld superplume is an exciting proposition. If substantiated by geochronology, this would link formation of one of the largest and most diversified metallogenic provinces in southern Africa (BIC^8^) to the energy trigger for world-class high-grade iron mineralisation in the Hamersley Province, where older sedimentary iron formations are continuously mineralised with localised high-grade hypogene iron deposits over a strike length of hundreds of kilometres^53^. Interestingly, synchronous ~ 2.06 Ga orthomagmatic Ni-Cu-PGE mineralisation also occurs at Kevitsa^54^ and Mirabela^55^, in the Baltica and Sao Francisco cratons, respectively. However, given the poor constraints on the paleogeographic relationship among the Baltica, Sao Francisco and Kaapvaal lithospheric blocks at ~ 2.06 Ga, it is not possible to ascertain whether the genesis of these magmatic systems, which are generally associated with mantle plume activity^55,56^, may also be related to the Bushveld superplume or simply reflect intense and global mantle plume activity at that time. The enhanced footprint of the Bushveld event that is demonstrated in this paper provides additional support for the idea that it had a major global environmental impact. In fact, the Bushveld event has been previously linked with the end of the major Lomagundi–Jatuli carbon isotopic excursion and has also been proposed to represent an appropriate proxy for the Rhyacian-Orosirian time boundary^57,58^.

## Conclusions

The geochronological results and 3D thermochemical modelling presented here support the hypothesis that a widely-dispersed ~ 2.06 Ga Proterozoic alkaline province in the eastern Yilgarn Craton of Western Australia represents a magmatic manifestation associated with the Bushveld superplume. Such an event has the capacity to generate melt fractions of ~ 5% at the lithosphere-asthenosphere boundary, which are required for the genesis of alkaline ultramafic magmatism, at distances between 2300 and 5500 km. Coupled constraints from geochronology, plume temperature excess and plume dynamics theory limit the plume diameter in the lower mantle to between 650 and 800 km, and its extent of surface melting to within a radius of 3000–4000 km. Impingement of this superplume may have driven the break-up of the Vaalbara Craton to form the now-separate Kaapvaal and Pilbara cratons. We also suggest that this plume event may have promoted a period of continental extension in the Pilbara Craton^51,59^, with widespread observed and inferred magmatism and hydrothermal activity leading to the formation of the world-class Hamersley Province ore deposits shortly after 2.06 Ga.

The impingement of superplumes can generate far-field effects, which indelibly modify the architecture and composition of lithospheric plates, including those separated by ocean basins at the time of plume impingement. Although this work is limited to the Bushveld and its products, the occurrence of several large plumes documented throughout the history of the planet suggests that many features observed in the continental lithosphere, such as the presence of magmatic provinces as well as intra-continental basins, could represent the lasting scars of other, perhaps less well-known superplume events^60^. In addition, the recognition of distal expressions of plume head magmatic activity in the form of alkaline magmatic provinces may indicate that other Proterozoic alkaline systems may also be related to mantle plumes centred beneath Archean lithospheric blocks that have long since been rearranged. Such alkaline provinces may now be orphaned from their plume origins by later rifting events and the dispersal of continental blocks.

## Supplementary information

Supplementary Information.
