# Supplementary material for: Bushveld superplume drove Proterozoic magmatism and metallogenesis in Australia
Source: Sci Rep. 2020 Nov 12;10:19729. doi: 10.1038/s41598-020-76800-0 (PMC7665188; doi:10.1038/s41598-020-76800-0)
Supplement: Supplementary file 1 — Supplementary Information. [file 41598_2020_76800_MOESM1_ESM.docx]

**Supplementary Material for "Bushveld super plume drove Proterozoic magmatism and metallogenesis in Australia"**

Marco L. Fiorentini^1*^, Craig O'Neill^2^, Andrea Giuliani^3,4^, Eunjoo Choi^1^, Roland Maas^4^, Franco Pirajno^1^, Stephen Foley^2^

^1^School of Earth Sciences, University of Western Australia, 35 Stirling Hwy, Crawley, WA6009 Australia

^2^Department of Earth & Planetary Science, Macquarie University, Sydney, NSW, 2109, Australia

^3^Institute of Geochemistry and Petrology, Department of Earth Sciences, ETH Zurich, Clausiusstrasse 25, 8092 Zurich, Switzerland

^4^School of Earth Sciences, University of Melbourne, Parkville VIC 3010, Australia

*Corresponding author: Marco L. Fiorentini (marco.fiorentini@uwa.edu.au)

**Geological Setting**

The Mt. Weld carbonatite complex and Melita ultramafic lamprophyre cluster (Supplementary Fig. 1) are located in the Eastern Goldfields Superterrane (EGS) of the Yilgarn Craton, Western Australia. From southwest to northeast, the Yilgarn Craton comprises the Kalgoorlie, Kurnalpi, Burtville and Yamarna terranes^1,2^. The Kalgoorlie Terrane contains 2.71-2.69 Ga tholeiitic and komatiitic units and the 2.69-2.66 Ga felsic volcaniclastic units^3,4^. The Kurnalpi Terrane consists of the 2.72-2.69 Ga mafic volcanic rocks, calc-alkaline complexes with 2.69-2.68 Ga bimodal rhyolite-basalt and calc-alkaline complexes^2,3^. The Burtville Terrane has 2.96-2.77 Ga successions of intermediate and felsic volcanic rocks and associated mafic-ultramafic rocks^5,6^. Farther to the east, the Yamarna Terrane (2.72-2.68 Ga) is separated from the older Burtville Terrane by the Yamarna Shear Zone, which has lithological and temporal affinities with the Kurnalpi and Kalgoorlie Terranes^5,6^.

Craton-wide felsic magmatism occurred from 2.65 to 2.62 Ga, leading to the cratonisation of the Yilgarn Craton into its current form and size^1,7,8,9^. Subsequently, during the Proterozoic eon multiple broad-scale magmatic events occurred within the Yilgarn Craton as well as along its margins. Aside the widespread ~2.05 Ga alkaline magmatic province that is the focus of this study^10^, the Widgiemooltha mafic dyke swarm was emplaced across the craton at ~2.4 Ga^11,12,13^. Along the southern, western and north-western margins of the Yilgarn Craton, magmas associated with the 1.21 Ga Marnda Moorn Large Igneous Province (LIP) were emplaced as widespread mafic dyke swarms. At ~ 1.07-1.065 Ga, the northern Yilgarn Craton was intruded by a series of mafic intrusions of the Warakurna LIP^14^.

The present-day lithosphere beneath the EGS is thinner than underneath the western part of the Yilgarn Craton, based on seismic tomography constraints^15,16^ as well as isotopic studies^17,18^. This architecture may have been conducive to focussing of distal ~2.05 Ga Bushveld-related alkaline magmatism in the EGS relative to adjacent lithospheric blocks, as discussed in the main text of this study.

**
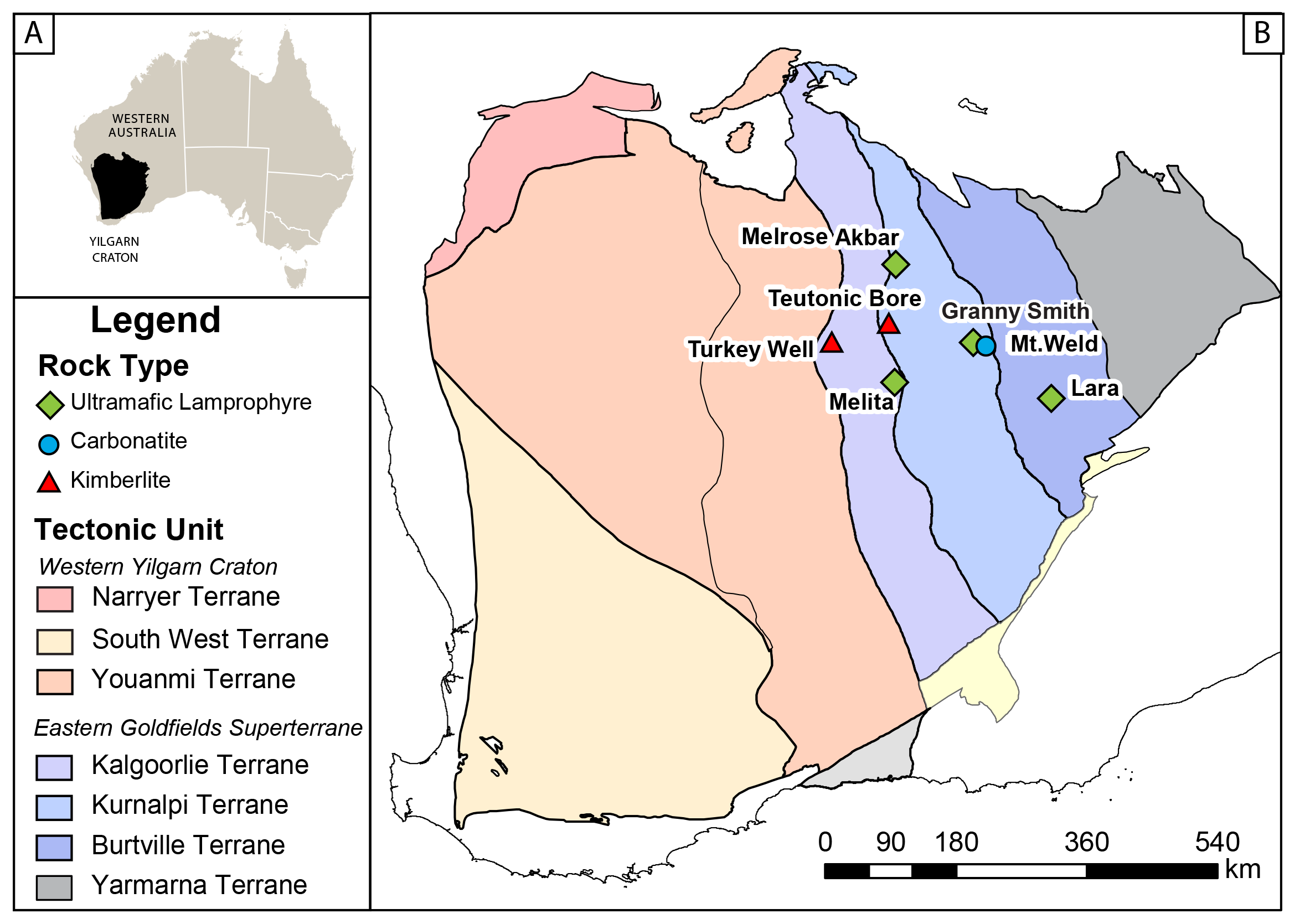
**

Supplementary Fig. 1 (A) Location of the Yilgarn Craton in Australia. (B) Simplified geological map of the Yilgarn Craton. The figure shows the distribution of different types of alkaline magmas, including the Melita ultramafic lamprophyre and Mt. Weld carbonatite that are investigated in this study. Modified from Choi et al^19^. This figure was created in Adobe Illustrator (www.adobe.com).

**Petrography and selected mineral chemistry**

Two samples from the Mt. Weld carbonatite and one specimen from the Melita ultramafic lamprophyre were selected for geochronological analyses. Major and minor element compositional analyses of mica and apatite in these samples were acquired on a JEOL JXA8530F electron microprobe at the Centre for Microscopy, Characterisation & Analysis (CMCA), The University of Western Australia. Trace element concentrations of apatite were analysed by LA-ICP-MS at the University of Western Australia, following the analytical protocol in Hammerli et al^20^.

***Mt. Weld carbonatite***

The Mt. Weld carbonatite can be classified as a sövite based on the abundance of calcite relative to other carbonate minerals^21,22^. The examined samples consist of calcite (> 50 vol. %), magnetite, phlogopite, apatite, pyrochlore and ilmenite (in order of decreasing abundance), with sulfides observed in some samples. Rock textures vary from massive and coarse-grained, indicating intrusive emplacement, to distinctly porphyritic, which may reflect shallower emplacement for some units.

Two specimens were selected for mica Rb-Sr and apatite U-Pb age dating. Sample 128211 has a porphyritic texture with phenocrysts of apatite and phlogopite in a groundmass of dominant calcite with lesser phlogopite (locally chloritised), magnetite and ilmenite. Phlogopite occurs as euhedral and subhedral grains up to 700 μm in length, and reaches 15 vol. % in abundance. Phlogopite exhibits concentric zonation, especially the phenocrysts, and contains Al_2_O_3_, FeO_T_, and MgO contents between 13.4-17.1 wt.%, 6.8-9.2 wt.%, and 19.2-21.6 wt.%, respectively (Supplementary Table 1). Phenocryst core compositions are higher in TiO_2_ and FeO_T_ than the phenocryst rim counterparts as well as groundmass grains, but contain lower Al_2_O_3_ and BaO contents. Apatite phenocrysts and groundmass grains occur as rounded or tapering prismatic crystals, with modal abundances of ~5 vol.%. Apatite phenocrysts are up to 900 µm in size and display cores overgrown by colloform rims that contain abundant calcite and phlogopite inclusions. The composition is typical of fluorapatite with low concentrations of REE and other minor elements in the cores, whereas the rims contain considerably higher REE contents (La_2_O_3_ + Ce_2_O_3 +_ Nd_2_O_3_ ≤ 2.5 wt.%; Supplementary Table 2). These compositions are broadly within compositional range of apatite in global carbonatites^23^.

The other sample CH04-5 employed for geochronology is a coarse-grained (intrusive) carbonatite characterised by massive texture with abundant calcite, apatite, tetraferriphlogopite, magnetite, and ilmenite. Tetraferriphlogopite grains (200-500 µm) exhibit euhedral shapes, and do not show zonation. Extremely low Al_2_O_3_ contents (less than 3.5 wt.%) and elevated FeO_T_ (16.8, and 17.4 wt.%; Supplementary Table 1) concentrations are typical geochemical features of tetraferriphlogopite in carbonatites (Lee et al., 2003; Reguir et al., 2009). Fluorapatite is the most abundant phase in the intrusive carbonatite CH04-5 beyond calcite, reaching 15 vol.%. Apatite grains show tapered rod- or pill-like shape, and vary in size between 70 and 300 µm. Apatite grains in sample CH04-5 are unzoned, and have compositions similar to the cores of fluorapatite phenocrysts in sample 128211 (Supplementary Table 2).

***Melita-2 ultramafic lamprophyre***

The examined sample is characterised by a porphyritic texture, containing phlogopite and completely serpentinised olivine phenocrysts in a groundmass of dominant clinopyroxene, partly chloritised phlogopite, serpentine, calcite, apatite and magnetite. Although samples from the Melita cluster were previously classified as kimberlites^10^, the abundance of clinopyroxene in the groundmass and composition of phlogopite (see below) indicate that these rocks are better classified as ultramafic lamprophyres^24,25^.

Phlogopite occurs as both phenocrysts (up to 600 μm) and groundmass (less than 100 μm) phases. Phlogopite grains display concentric zoning, but the compositional differences between cores and rims are not systematic. Melita phlogopite contains TiO_2_, Al_2_O_3_, FeO_T_, MgO and BaO concentrations of up to 4.0 wt.%, 13.1 wt.%, 6.7 wt.%, 20.7 wt.%, and 0.5 wt.% respectively (Supplementary Table 1). These compositions are similar to those found in global ultramafic lamprophyres^26,27^, but they are relatively enriched in TiO_2_ and depleted in BaO contents, unlike phlogopite in kimberlites^24^.

Supplementary Table 1*.* Representative major and minor oxide compositions (wt.%) of selected phlogopite grains from the Mt. Weld carbonatite and Melita-2 ultramafic lamprophyre.


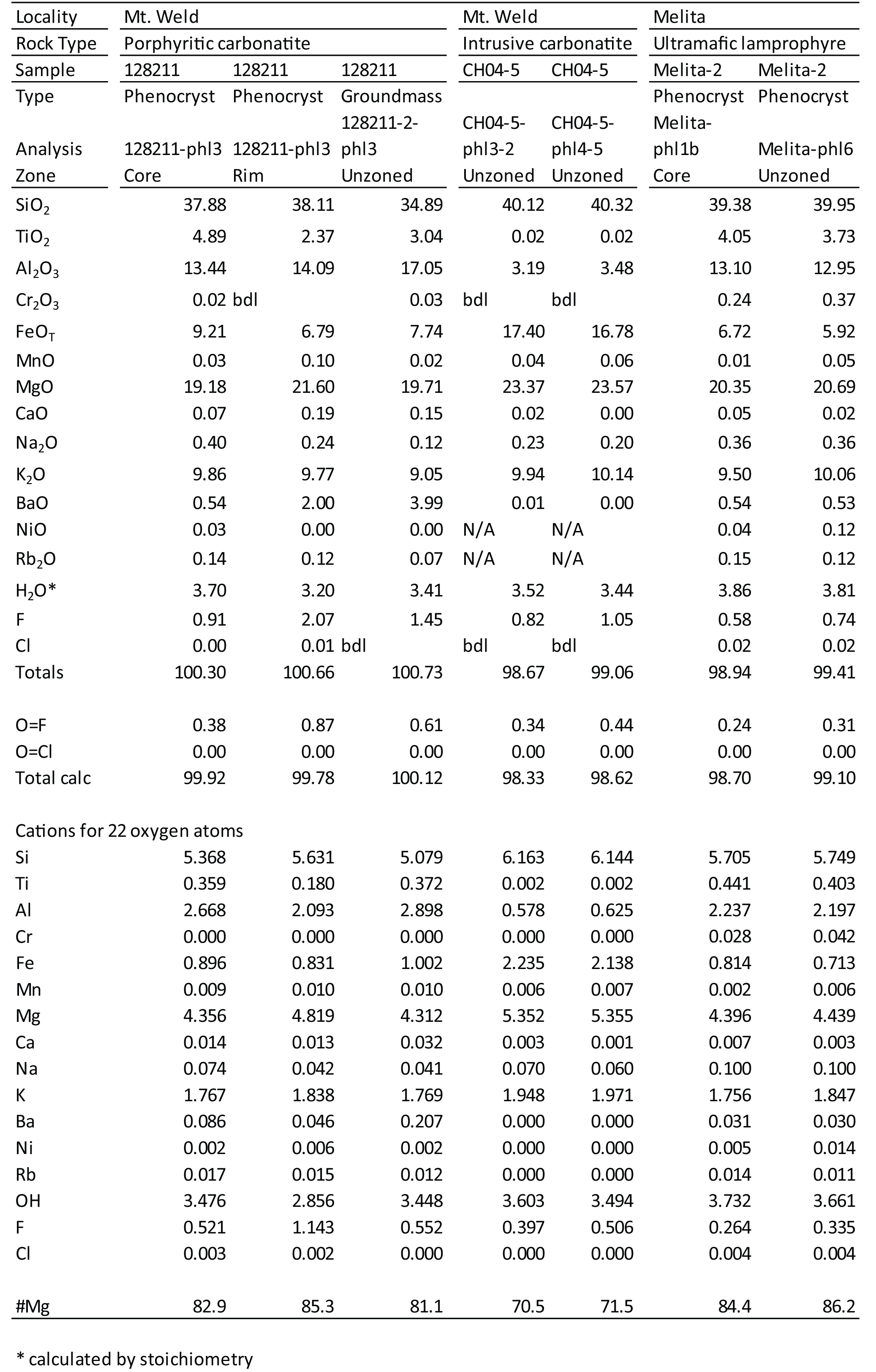


Supplementary Table 2*.* Representative major and minor oxide compositions (wt.%) of selected apatite grains from the Mt. Weld carbonatite.


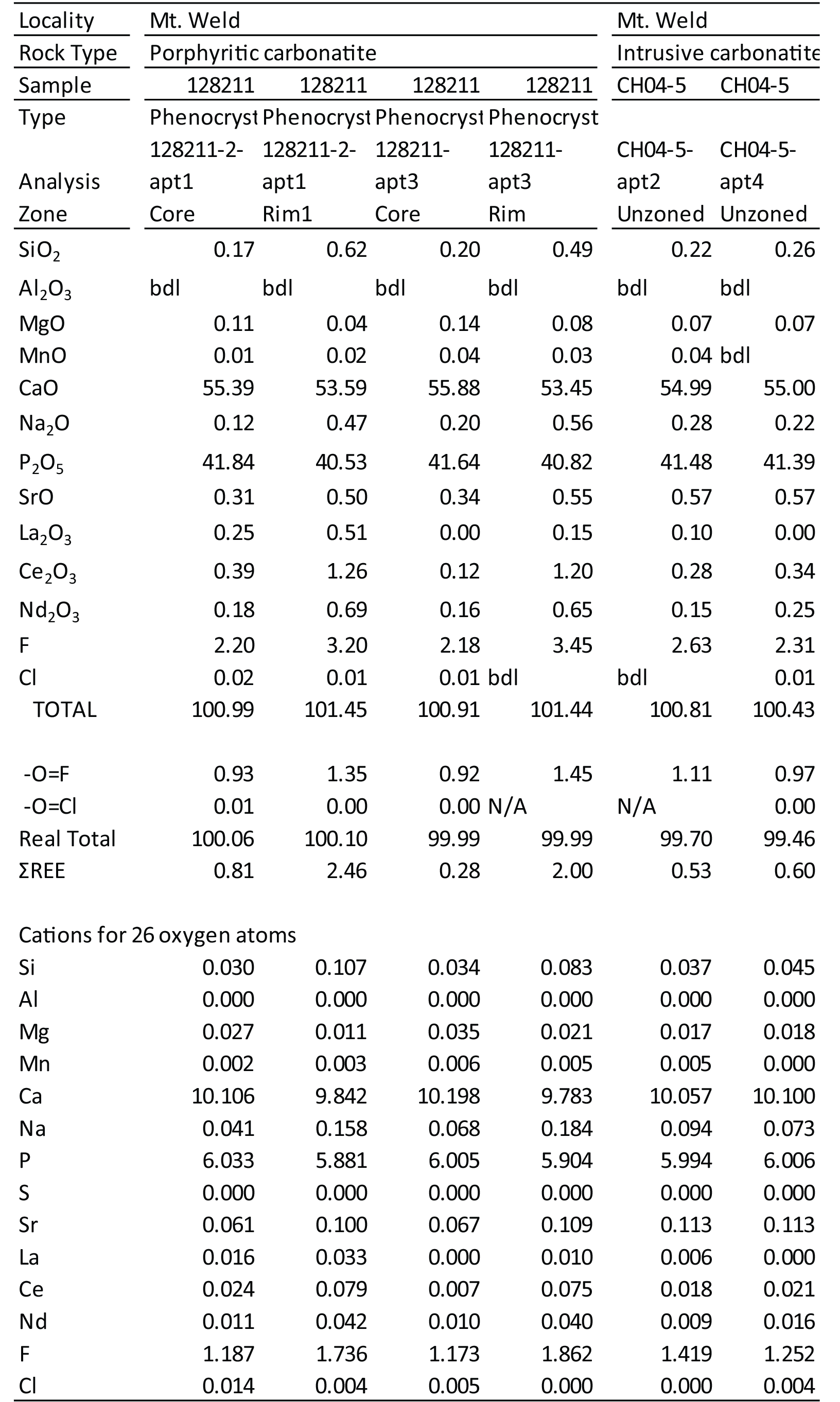


**3. Rb-Sr dating of mica**

***Methods***

Rb-Sr dating of mica from the Mt. Weld carbonatite and the Melita ultramafic lamprophyre was carried out at the University of Melbourne, using procedures adapted from Maas et al^28^ and Yaxley et al^29^. Mt. Weld sample CH04-5 represents an intrusive carbonatite with large tetraferriphlogopite grains, whereas porphyritic sample 128211 from Mt. Weld and Melita-2 from Melita ultramafic lamprophyre contain phlogopite as both phenocryst and groundmass phases. Multiple fractions of phlogopite (3-22 mg) from each sample were cleaned with hot distilled water, followed by mild acid leaching (2M HNO_3_, 20^o^C, 2 min) of some fractions. The leaching dissolves Sr-rich calcite impurities and produces a phlogopite residue with higher Rb/Sr. The nitric acid leach solutions (plus multiple rinses with distilled water) were stored for later analysis. Following dissolution in HF-HNO_3_, the phlogopite fractions were equilibrated with a ^85^Rb-^84^Sr spike; the nitric acid leach solutions for five of the Mt. Weld and one of the Melita fractions were also spiked. Sr and Rb were extracted using a combination of Eichrom Sr resin and AG50-X8 cation resin. Procedural blanks were <0.1 ng and blank corrections were negligible in all cases, even for the leach fractions with low Rb/Sr (Rb sample/blank ratios >370). All isotopic analyses were done on a Nu Plasma MC-ICPMS. Strontium isotope ratios were corrected for interference from Kr and Rb (usually negligible, but reaching ^85^Rb/^86^Sr ~ 0.001 in rare cases; measured - i.e. spiked - ^85^Rb/^87^Rb is used rather than the natural ratio) and internally normalised to ^88^Sr/^86^Sr = 8.37521 using an online iterative spike stripping-normalization routine. Corrected ^87^Sr/^86^Sr ratios are reported relative to SRM987 = 0.710230 and have an external precision (2sd) of 0.000040. Rubidium isotope dilution analyses were doing using Zr-doping. ^87^Rb/^86^Sr determined by isotope dilution has an external precision of ±0.5% (2sd).

***Rb-Sr isotope results for standards***

Two different Rb-Sr spikes were used in this study: a ^85^Rb-^84^Sr (with high-purity ^84^Sr, >99%) mix suitable for materials with high Rb/Sr (^87^Rb/^86^Sr >10), and another ^85^Rb-^84^Sr mix (with low-purity ^84^Sr, ~82%) optimized for materials with lower Rb/Sr. This was necessary to avoid under/overspiking of the diverse samples handled in this study. In an effort to confirm that results obtained with these two different spike mixtures are identical, replicate analyses using the two spikes were performed for the GLO-1 glaucony standard, the SRM607 K-feldspar standard, and one of the Melita phlogopite sample solutions which was gravimetrically split and then spiked. In all cases, the Rb-Sr isotope results and calculated model ages for the respective replicates were indistinguishable: the two analysed fractions of GLO-1 glauconite (ca. 90 Ma; Odin^30^) yielded model ages of 90.42 ± 0.49 and 90.23±0.48 Ma (with an assumed initial ^87^Sr/^86^Sr of 0.7074), those of NIST SRM607 feldspar (ca. 1430 Ma^31,32^) yielded 1431.9±7.0 and 1431.6±7.0 Ma (with an assumed initial ^87^Sr/^86^Sr of 0.710), and Melita phlogopite 1 (MPhl1) yielded 2059±10 and 2061±10 Ma (with an assumed initial ^87^Sr/^86^Sr of 0.706). Furthermore, a mineral isochron for hornblende (2 fractions spiked with the low-Rb/Sr spike) and biotite (3 fractions spiked with high-Rb/Sr spike) from the Mt. Dromedary monzonite, source of the GA1550 biotite ^40^Ar-^39^Ar standard (e.g., Phillips et al., 2017), yielded an age of 99.05±0.33 Ma (MSWD = 0.066, initial ^87^Sr/^86^Sr = 0.70451 ± 0.00005, 95% CL, n = 5). All standard results are consistent with reference ages (adjusted to the current Rb decay constant, 1.397x10^-11^/yr^33^) and indicate a high degree of accuracy and precision in the Rb-Sr isotope work presented here. Isochron and model ages were calculated using the ISOPLOT software^34^, with input errors of 0.5% (2sd) for ^87^Rb/^86^Sr and 0.01% for ^87^Sr/^86^Sr.

**Rb-Sr isotope results for phlogopite unknowns**

***Mt. Weld carbonatite***

Unleached tetraferriphlogopite from sample CH04-5 has 785 ppm Rb and Sr is very high (1151 ppm, Supplementary Table 3). Brief contact with ~13% HNO_3_ removed >90% of the Sr in residues MW1.3R and 1.4R without changing the Rb concentrations, suggesting that Sr is largely held in Sr-rich and soluble impurities (probably calcite). The resulting higher-purity phlogopite residues are highly radiogenic (^87^Sr/^86^Sr = 1.49 - 1.61). By contrast, residue MW1.2R, picked from the same concentrate, still has >400 ppm Sr after leaching, with Rb (and Ba, unpubl. data) being much lower than in the bulk tetraferriphlogopite and the other residues. As a result, the Rb-Sr system of this residue is less radiogenic (^87^Sr/^86^Sr = 0.73). The nitric acid leachates are invariably rich in Sr (>1 wt% Sr) and have very low Rb/Sr and measured ^87^Sr/^86^Sr is 0.7024-0.7029.

Rubidium concentrations in phlogopite from sample 128211 (743 ppm) resemble those in CH04-5 but Sr concentration is far lower (153 ppm). Nevertheless, the acid leachates contain large amounts of Sr suggesting the content of calcite impurities in the 3 analysed phlogopite fractions varied strongly. The residues and unleached phlogopite are all very radiogenic (measured ^87^Sr/^86^Sr = 1.05-1.34). The corresponding leachates are once again Sr-rich and unradiogenic (^87^Sr/^86^Sr = 0.7023-0.7031).

Various combinations of the Mt. Weld phlogopite Rb-Sr data points in the isochron diagram yield slopes equivalent to ages from 2054±7 to 2061±10 Ma (95% CL), with indistinguishable ranges in age and initial ^87^Sr/^86^Sr (0.7019-0.7022) for the intrusive and eruptive facies studied here (Supplementary Table 3). A regression for all 13 data points yields an age of 2058.2 ± 4.9 Ma (95% CL, MSWD = 1.9, initial ^87^Sr/^86^Sr = 0.70192 ± 0.00034; Figure 2A).

***Melita ultramafic lamprophyre***

Phlogopite in sample Melita-2 has 634-636 ppm Rb and 212-213 ppm Sr (n = 2; Supplementary Table 3), and thus a relatively high Rb/Sr ratio. Acid leaching of three further fractions removed substantial Sr without change to Rb contents (505-892 ppm). The resulting residues have variably higher Rb/Sr with ^87^Sr/^86^Sr of between 1.04-1.43. A single analysed leach solution is once again rich in Sr (2031 ppm) and has low Rb/Sr and ^87^Sr/^86^Sr (0.7046). A regression through all the 6 data points yield an apparent age of 2061±17 Ma (95% CL, MSWD = 4.7, initial ^87^Sr/^86^Sr = 0.7044 ± 0.0030; Figure 2B in main text).

**Apatite U-Pb dating**

***Methods***

Laser-ablation ICP-MS U-Pb isotope analyses of apatite grains from the Mt Weld carbonatites were carried out at the University of Melbourne, using a Resonetics Resolution 193 nm excimer laser ablation system interfaced with an Agilent 7700x quadrupole ICP-MS^34^. Apatite was analysed in two thin sections from porphyritic carbonatite sample 128211, and in one thin section from coarse-grained intrusive sample CH04-5. Clean areas, i.e. free of inclusions and away from cracks, in grains typically larger than 100 μm (in diameter) were selected for analysis. Targets were ablated for 30 s using a 60 μm laser spot size (repetition rate 5 Hz, fluence ~2.5 J/cm^2^), followed by a 15 second washout period; backgrounds were measured during 50 s breaks inserted after every 5^th^ ablation. The isotopes monitored were ^206-207-208^Pb, ^232^Th and ^235-238^U. Instrumental drift and downhole fractionation were corrected using the BR13 apatite (mean ^207^Pb/^206^Pb age = 2105.5 ± 6.3 Ma, 2sd, R Maas & A. Kennedy, unpubl. Data). The OD306 apatite (from Acropolis, Olympic Dam, South Australia; age = 1597 ± 7 Ma^35^) was used as secondary standard to assess data reproducibility and yielded an age of 1579 ± 4 Ma, within ~1% of the nominal age (Supplementary Table 5). Data reduction was done using the UncomPbine data reduction scheme of the Iolite software package^36,37^ and the results were evaluated using ISOPLOT and ISOPLOT-R^39.40^.

Supplementary Table 3. Rb-Sr isotope results for mica from the Mt. Weld carbonatite and Melita ultramafic lamprophyre.


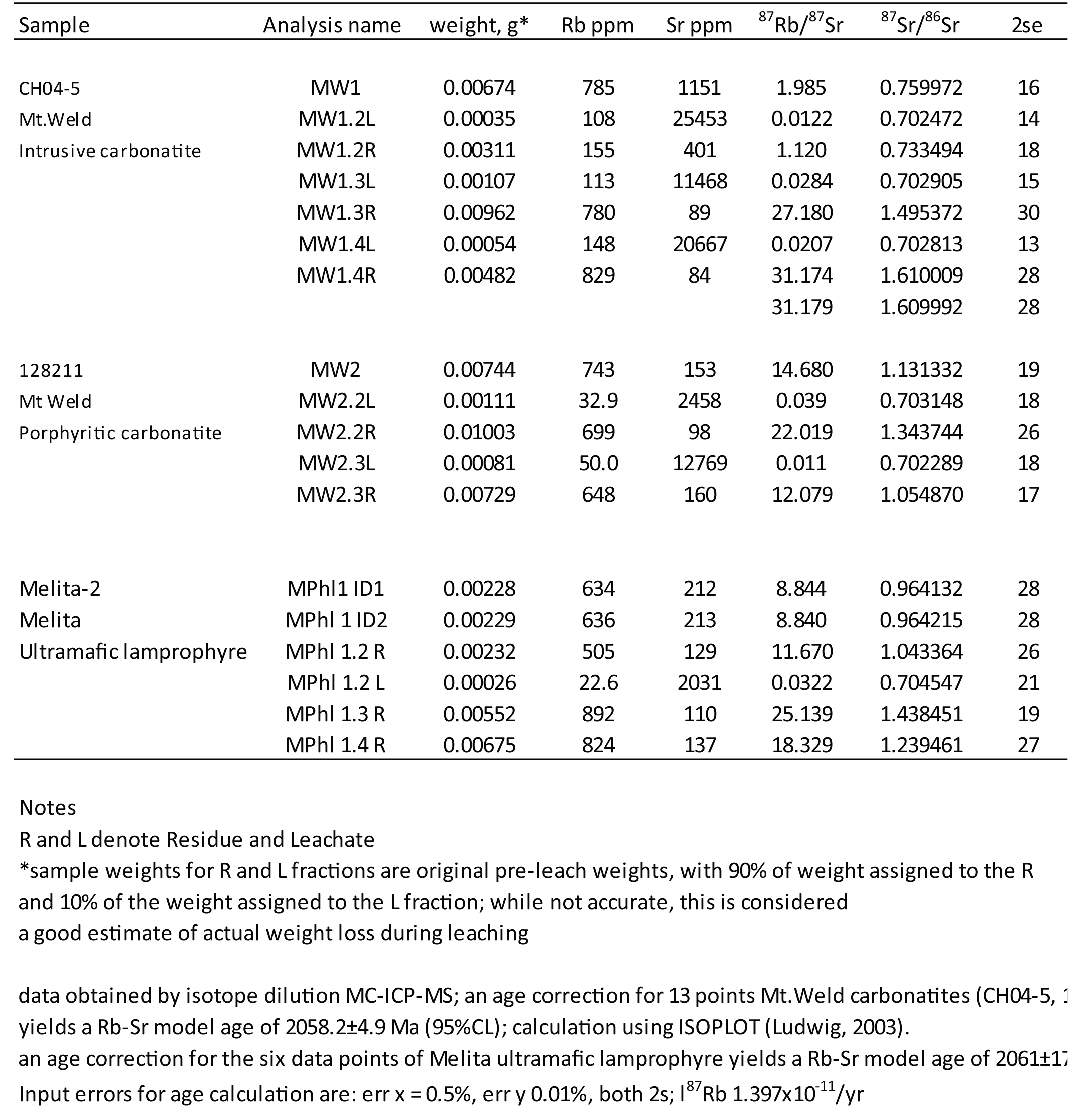


***Apatite U-Pb results***

Apatite grains in samples 128211 and CH04-5 contain 7-20 ppm Pb, 4-13 ppm U and 28-76 ppm Th with highly variable U/Pb and Th/U ratios (<1.5 and <22, respectively; Supplementary Table 4). ^207^Pb/^206^Pb also varies widely with similar ranges in the two samples (0.17-0.58, n = 46 in 128211; 0.21-0.65, n = 25 in CH04-5; Supplementary Table 5). This implies large variation in the ratio of radiogenic to initial Pb between ablation sites/grains. However, no systematic difference was observed between core and rim of large grains or with grain size. Regressions through the data yield lower intercept ages of 2059 ± 15 Ma (95% CL, n = 46; MSWD = 1.7) for sample 128211 (Figure 2C in main text). Dating of apatite in an intrusive carbonatite sample (CH04-5) from the same locality returned a somewhat older age of 2089 ± 13 Ma (n = 25; MSWD = 2.4; Supplementary Fig. 2). The reason for this difference is unclear and cannot be due to analytical bias as the two samples were analysed in the same session. Likewise, this age difference cannot be attributed to insufficient sampling because apatite from both samples shows similar large spreads in ^238^U/^206^Pb as well as ^207^Pb/^206^Pb ratios, including indistinguishable initial ^207^Pb/^206^Pb compositions of 0.97 ± 0.02 and 1.01 ± 0.03, respectively. Initial ^207^Pb/^206^Pb of 0.97 ± 0.02 and 1.01 ± 0.03 are very close to the respective ^207^Pb/^206^Pb ratios on global Pb growth curves (e.g., 1.0084 ± 0.0019/0.0012 for 2059 ± 15 Ma on the growth curve of Stacey and Kramers^41^). Based on the similarity with phlogopite Rb/Sr ages for both Mt. Weld carbonatite and Melita ultramafic lamprophyre, we only consider reliable the younger apatite U-Pb age of porphyritic sample 128211.

Supplementary Table 4*.* Representative trace element contents (ppm) of apatite from the Mt. Weld carbonatite.


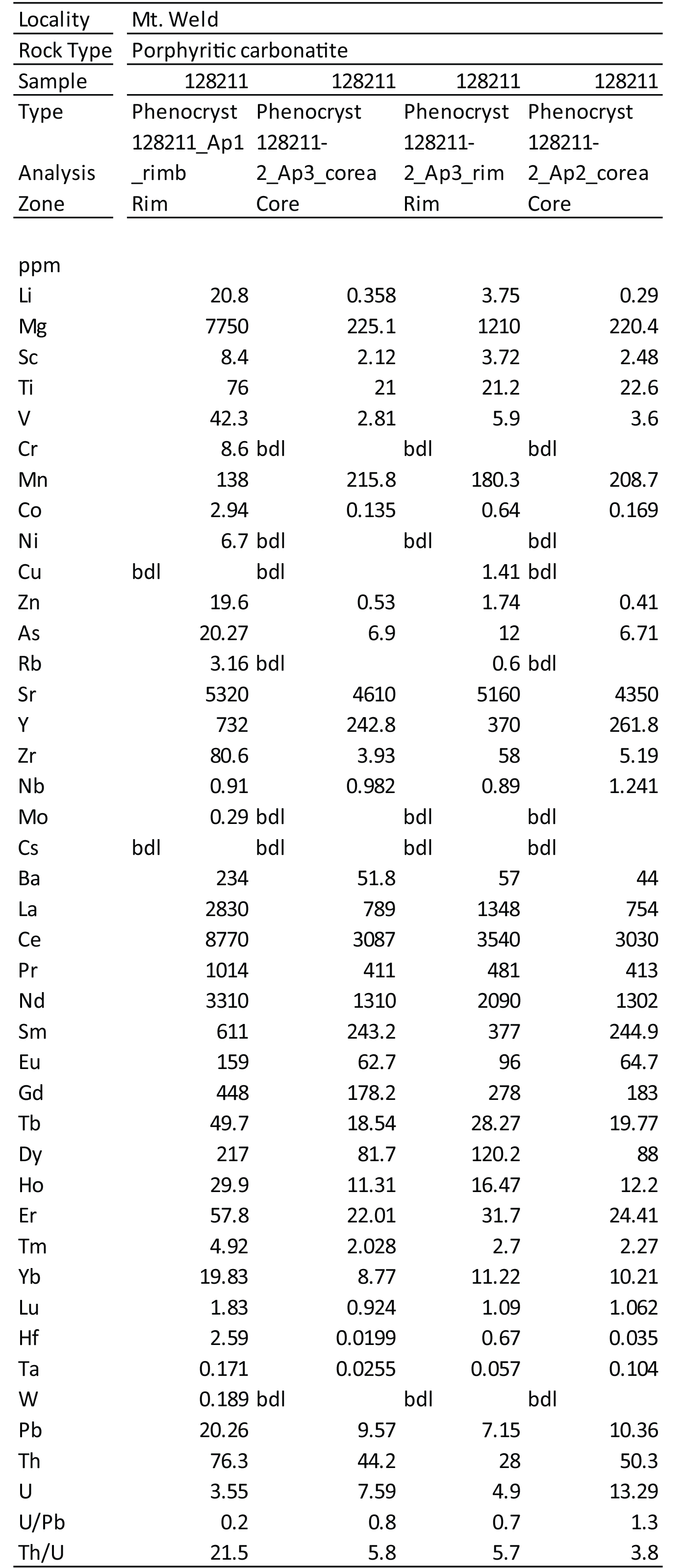


Supplementary Table 5. U-Pb isotope compositions of apatite grains analysed in situ by La-ICPMS.


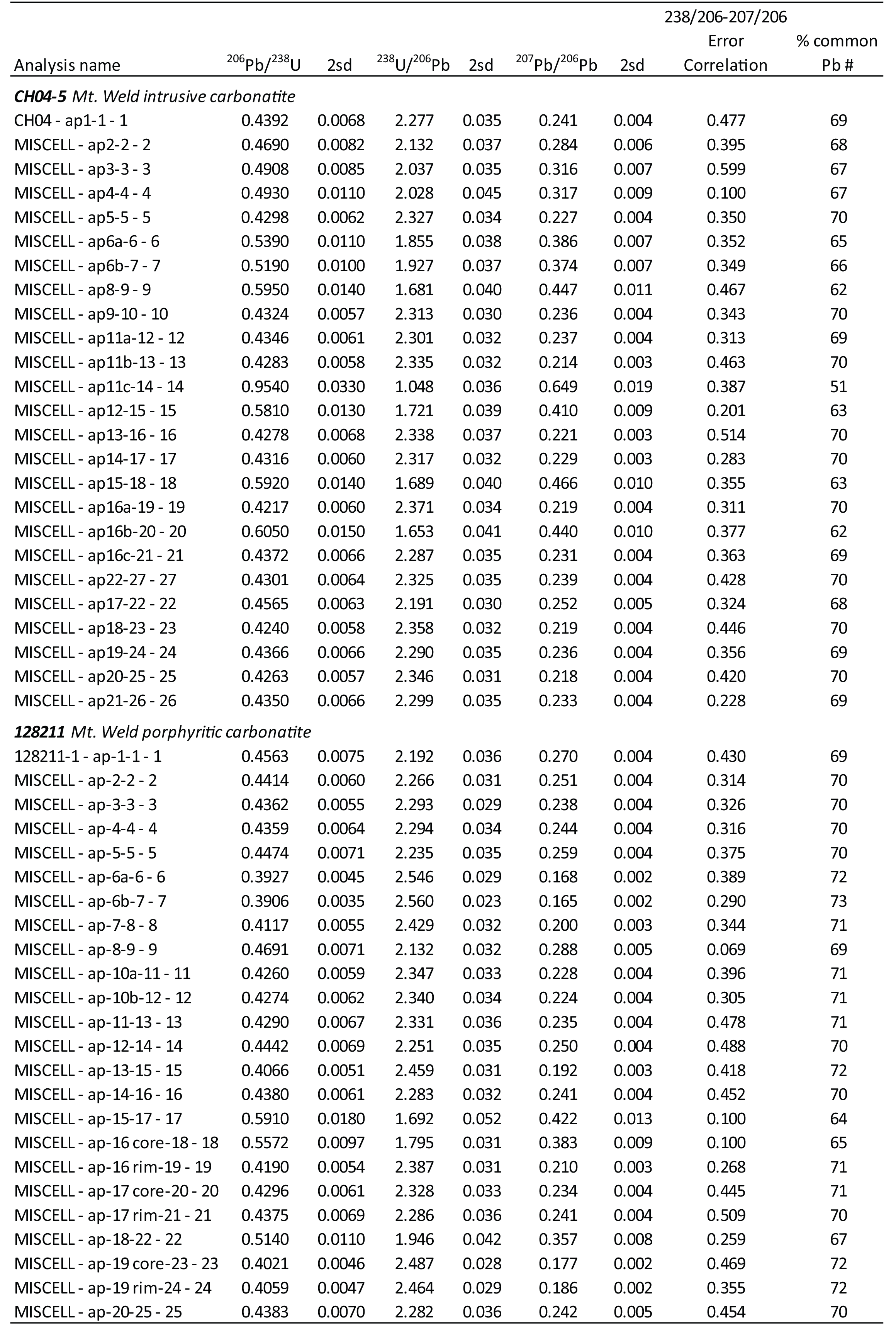


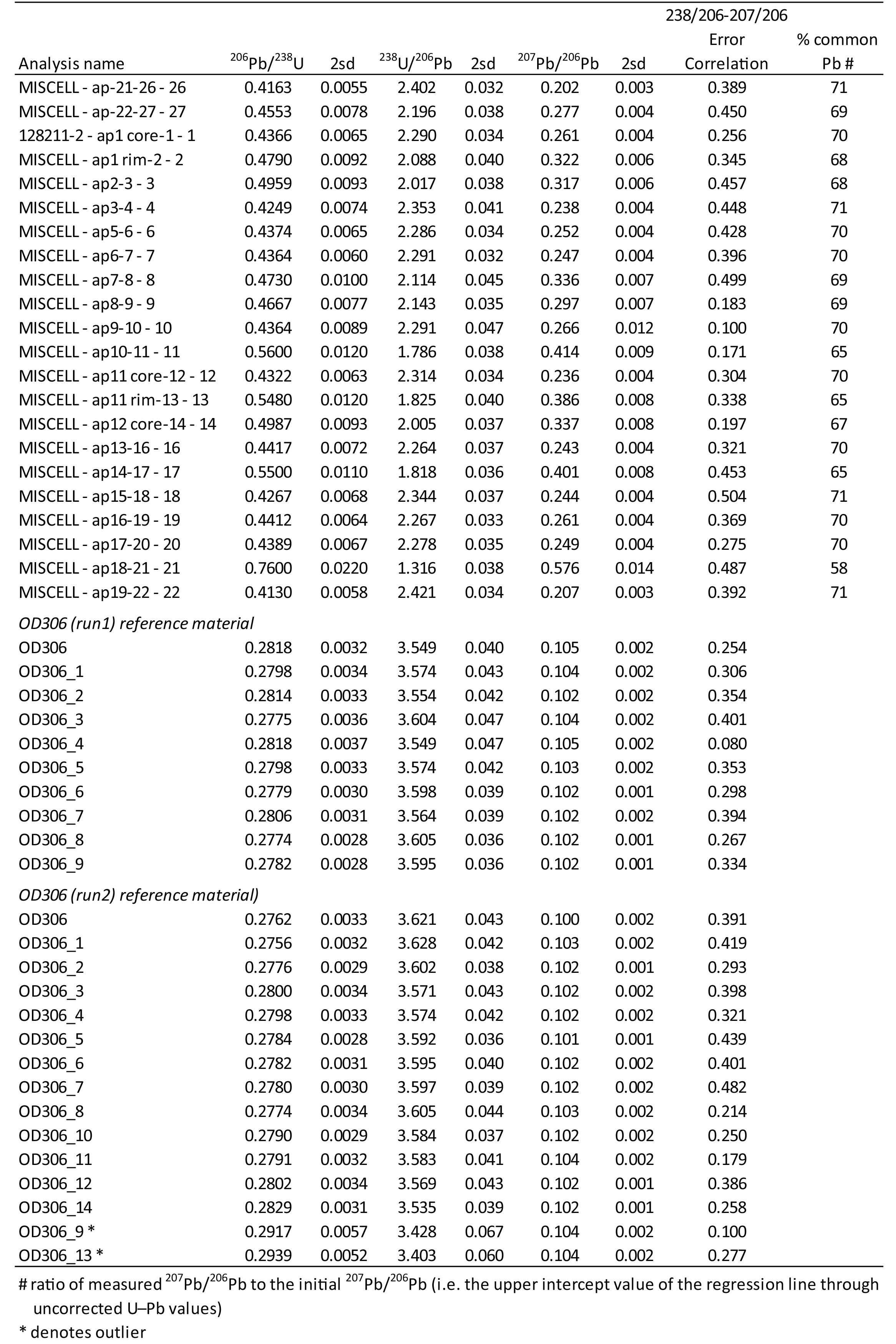


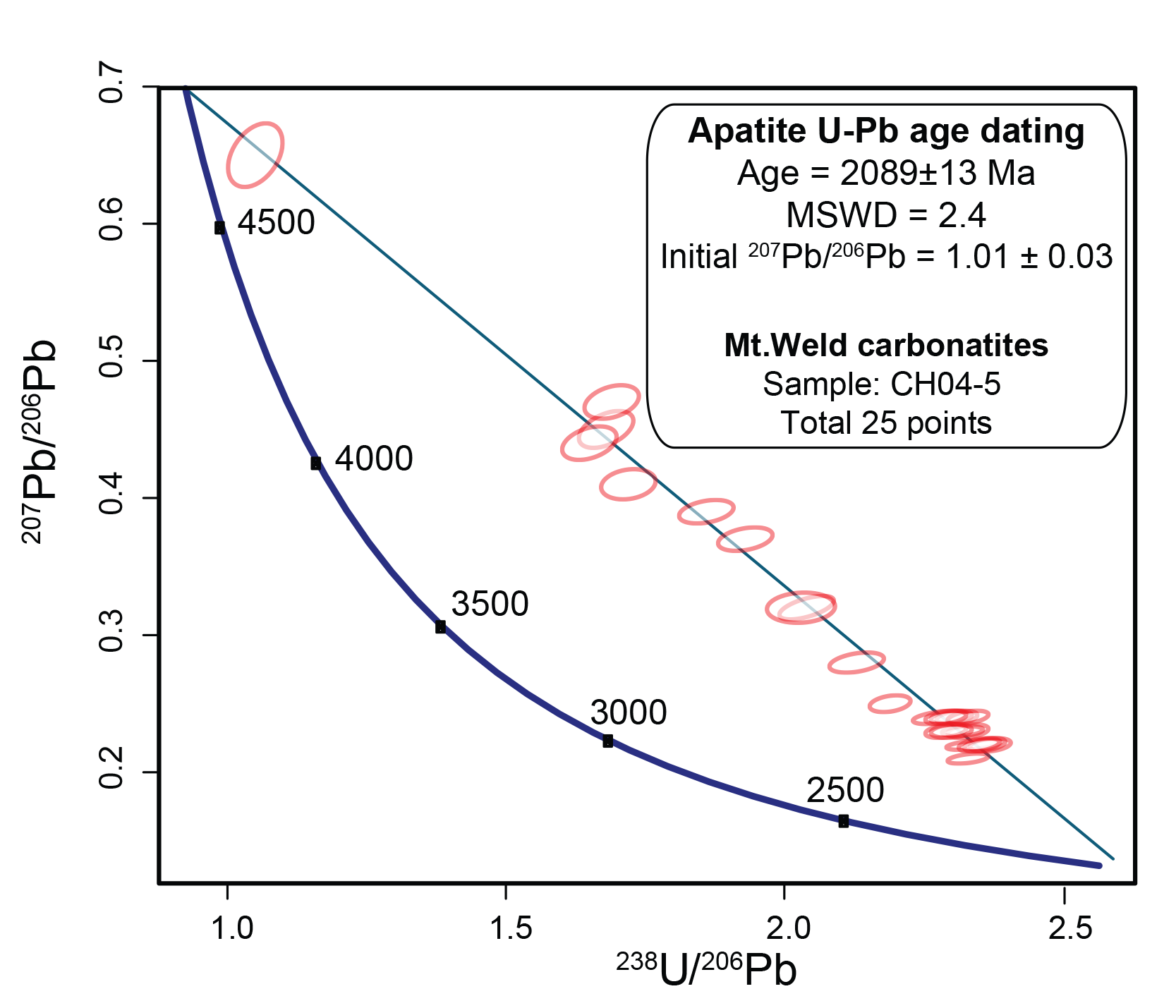


Supplementary Fig. 2 Tera-Wasserburg U-Pb isochrone diagram for apatite analysed by La-ICPMS from sample CH04-5. The isochron was generated using IsoplotR^51^ and the figure was created in Adobe Illustrator (www.adobe.com).

**Comparison of phlogopite and apatite ages with existing geochronology**

Existing estimates for the emplacement age of the Mt. Weld Carbonatite range from

2025 ± 10 to 2090 ± 10 Ma. The most widely-known radiometric age of 2025 ± 10 Ma is based on a Re-Os isochron that includes bulk rocks, ilmenite and spinel separates from samples of kimberlites, ultramafic lamprophyres and carbonatites across the Eastern Goldfield Superterranes^10^. A Pb-Pb isotope age of 2090 ± 10 Ma (based on 2 data points only) was reported in Nelson et al^42^. In addition, unpublished ages of 2021 ± 13 Ma (Rb-Sr, Collerson^43^) and 2064 ± 40 Ma (K-Ar, A. W. Webb, AMDEL) were quoted in Jaireth et al^44^. The Rb-Sr age of 2021 ± 13 Ma shifts to 2053 ± 13 Ma after adjustment to the updated Rb decay constant^34^ and is thus indistinguishable from the Rb-Sr and U-Pb apatite (for sample 128211) ages reported here.

Beyond the 2025 ± 10 Ma of Graham et al^10^, the only radiometric data available for the Melita ultramafic lamprophyre are those from White^45^. Phlogopite from Melita 01 yielded ^40^Ar/^39^Ar total fusion ages of 1935 ± 5 and 1935 ± 8 Ma while Rb-Sr data for phlogopite, strongly acid-leached phlogopite and the complementary leaching acid produced a scattered 3-point line with an age of 2096 ± 240 Ma (adjusted to updated Rb decay constant, MSWD 9.1) with an unrealistically low initial ^87^Sr/^86^Sr of 0.695 ± 0.032. While inconsistent, these ^40^Ar/^39^Ar and Rb-Sr results indicate a likely age around 2 Ga for the Melita ultramafic lamprophyre. We note that the Rb-Sr composition of the leached phlogopite fraction reported in White^45^ generates a model age of 2057±10 Ma for an assumed initial ^87^Sr/^86^Sr of 0.704±0.002, identical to the Rb-Sr phlogopite age reported in this study.

**Model information**

***Model setup***

We use the community code Aspect for our simulations, with a diffusion/dislocation creep rheology^46^. The code solves the general (vertical) compressible convection equations for mass, momentum, and energy conservation. The energy equation incorporates decaying radioactive heating sources, shear heating, and adiabatic heating.

Material properties such as density, heat capacity, thermal expansivity, and compressibility are calculated using lookup tables calculated by Perplex (<http://www.perplex.ethz.ch/>) using the thermodynamic database of Stixrude et al^47^ for a pyrolitic composition^48^. Other key parameters are shown in Supplementary Table 6.

We use a composite viscosity based on four deformation mechanisms: diffusion creep, dislocation creep, Peierls creep (which take an Arrhenius form), and yielding (which follows a Byerlee-style law^49^). The effective viscosity is based on a parallel law. The rheological parameters used in our models are shown in Supplementary Table 7.

Supplementary Table 6. Model parameters used in this study.


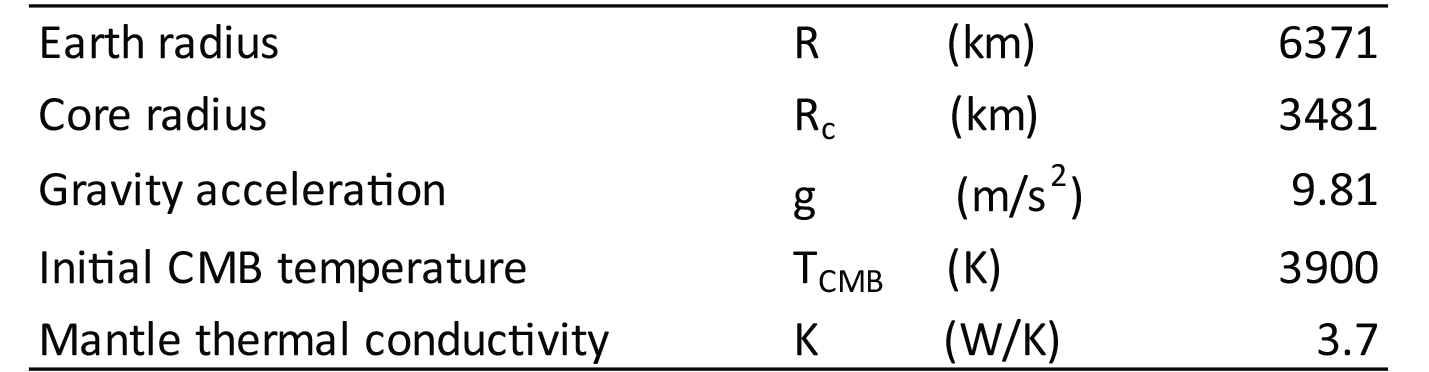


Supplementary Table 7. Rheological parameters used in this study.


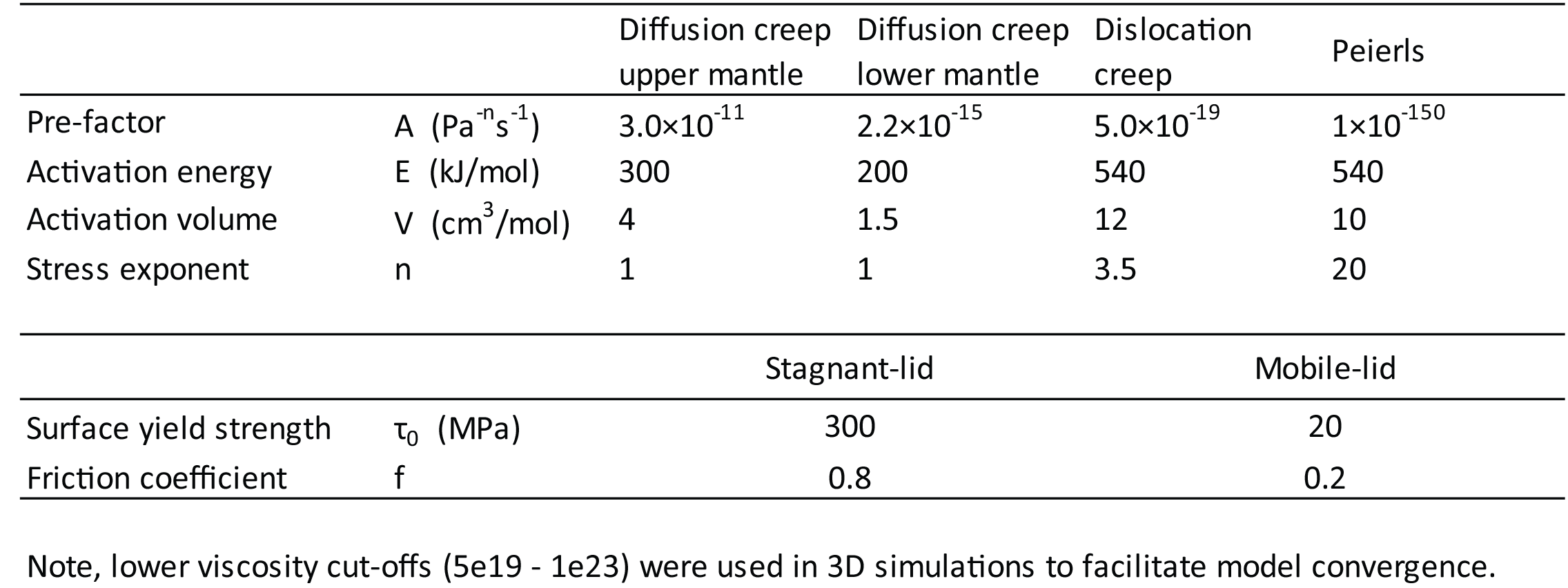


***Initial conditions***

We impose a sub-solidus initial temperature model shown in Supplementary Figure 3. The core temperature is set to 4200K and the surface temperature is 300K. The lithospheric thickness is initially set at 80km outside of the cratons, and the basal thermal boundary layer as 200km thick (variable: 40km-250km), with 100km amplitude perturbations imposed on this layer, with wavenumbers 2 and 3 in the lateral directions (variable, up to 8 + 8). Together these wavenumbers and basal thermal boundary thickness control the initial plume size, which we derive *a posteri*, and vary these parameters between simulations of different plume size. The cratons are imposed as 2 overlapping spherical compositional domains of radius 250km (and centers positioned at the surface), giving cratonic roots 250km thick. Cratonic mantle is assumed to be 100 times more viscous than ambient mantle, and 50kg/m^3^ less dense, due to dehydration and depletion^50^.


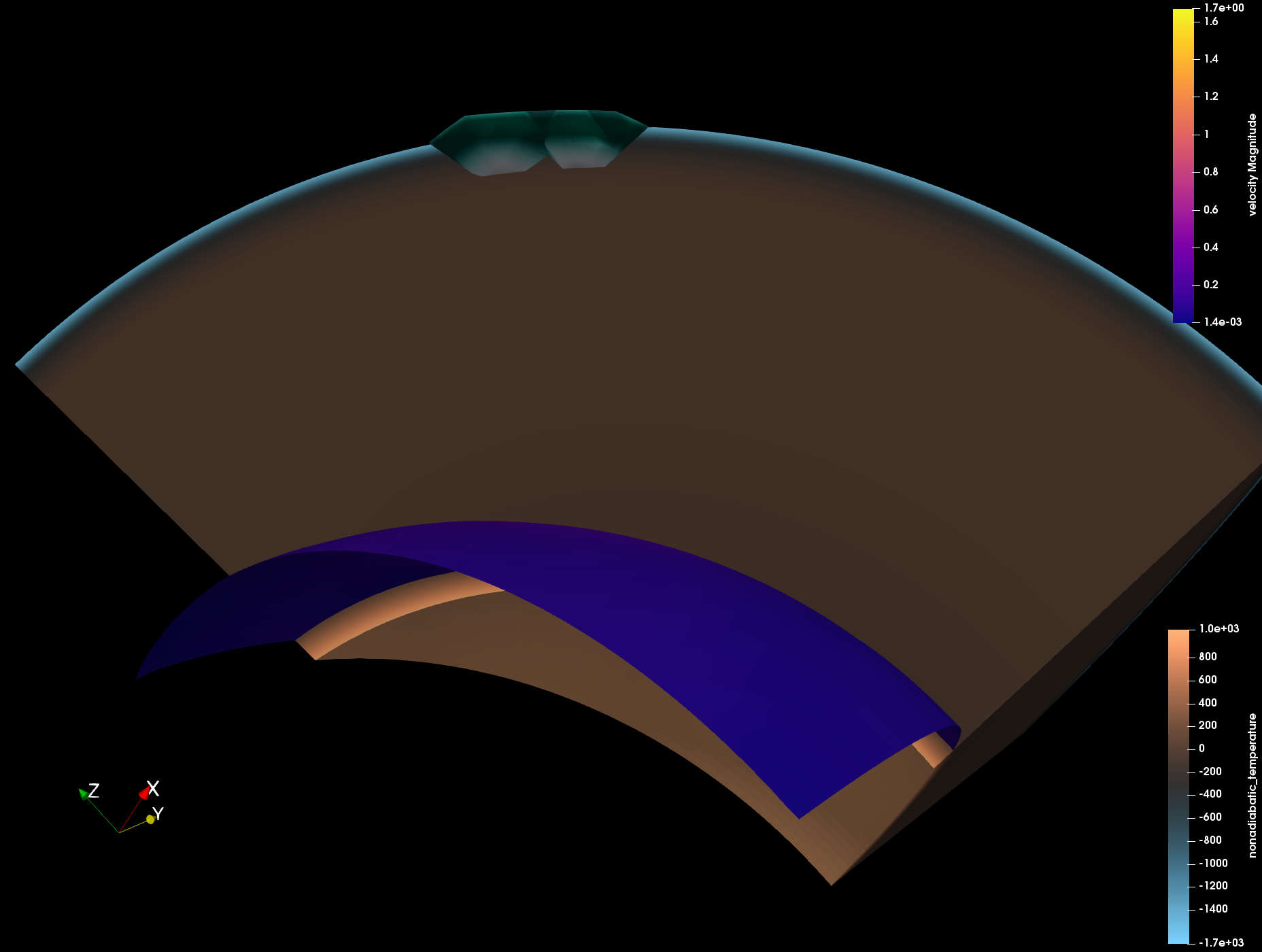


***
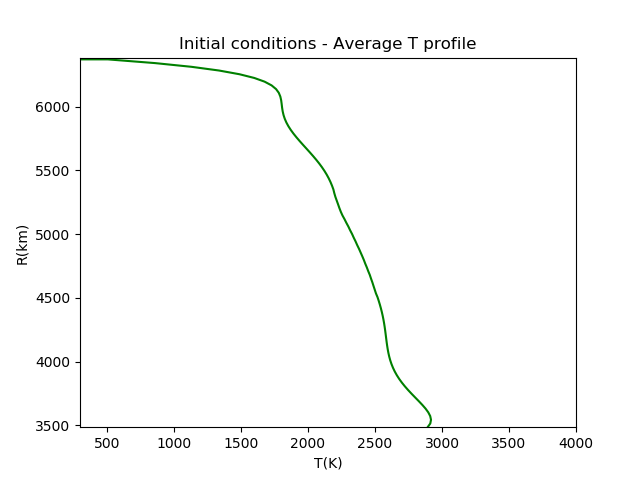
***

Supplementary Figure 3. a) Snapshot of initial conditions, showing imposed cratonic roots (green tones), a temperature isocontour of +200K above the adiabat (blue surface), and nonadiabatic temperatures (background slice colour). b) Initial temperature profile of the simulation. Simulation performed and figure created with with geodynamics code Aspect 2.0 (aspect.geodynamics.org).

***Lithospheric melting analysis***

We address the potential for lithospheric melting in producing alkaline magmatism. The following are 2D models, represented in the aggregation in Supplementary Figures 4-6. The first (Supplementary Fig. 3) shows the extent of mantle melting in the plume head, using the solidus of Stixrude et al^47^. Small fraction melts extent throughout most of the plume head. Figures 5 and 6 show mantle lithospheric melting using two solidii, a "reduced" mantle fluid-influenced solidus (Supplementary Fig. 5), and an "oxidised" fluid-influenced solidus (Supplementary Fig. 6). Lithospheric melting in these cases is powered by heat provided by the plume, and their lateral extent is broadly congruent with the plume head.

In both lithospheric melting cases, the furthest extent of lithospheric melting to produce alkaline melts coincides with the lateral extent of the plume, and is broadly congruent with the extent of mantle melting within the plume head. There is a trade-off in these models between the initial Proterozoic adiabatic gradient, the Core-Mantle boundary temperature, and the degree of melting observed in plumes (high values of the former result in large amounts of melt). However, the overall extent of plume influence depends primarily on its size and buoyancy (including temperature contrast), and thus is somewhat less sensitive to melt fraction than these variables.


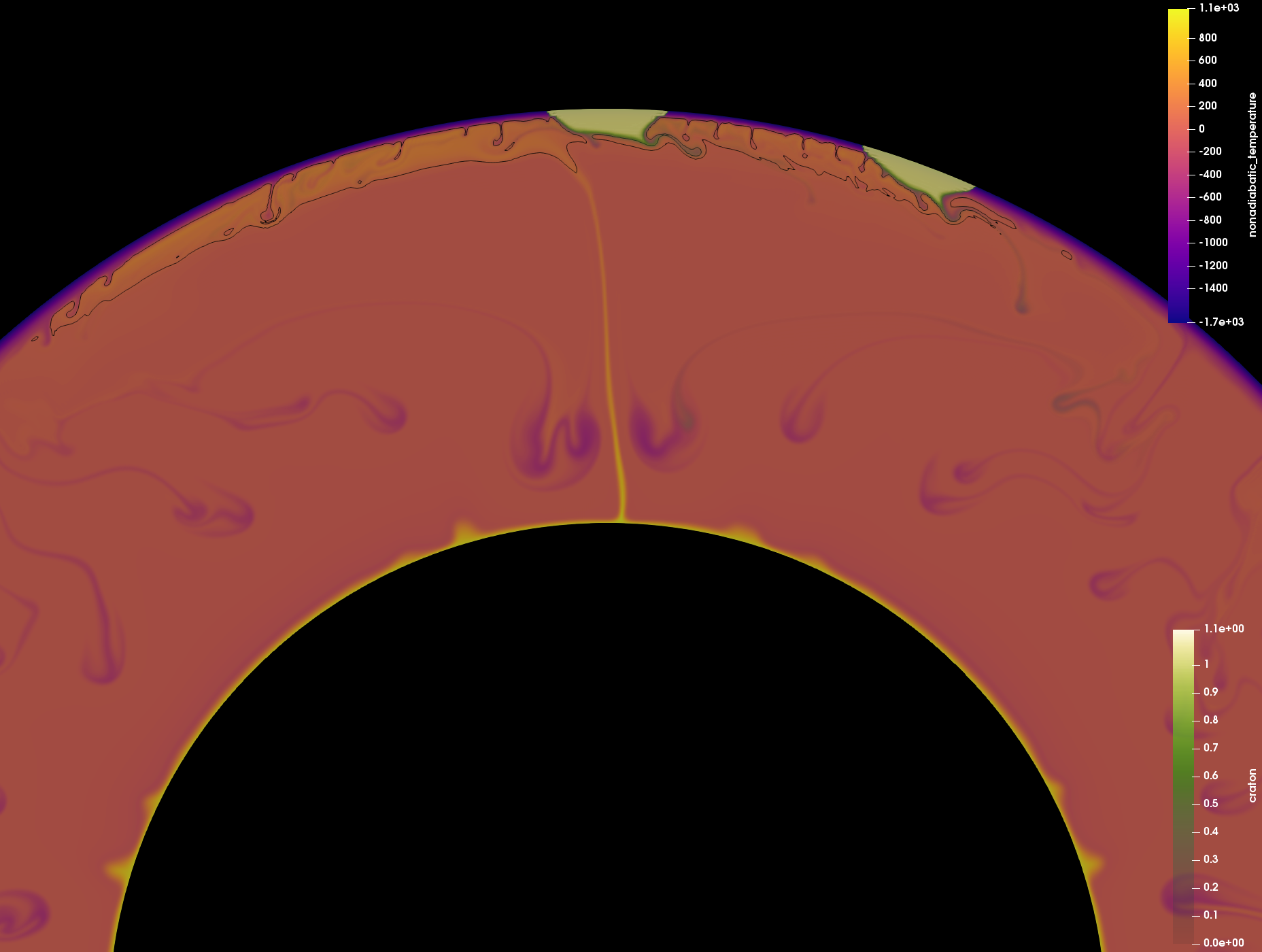


Supplementary Fig. 4. 2D model of mantle plume arrival beneath imposed lithospheric/cratonic architecture. The plume extends >>2000km, taking ~12 Myrs to move that distance laterally. Isocontour is for 2% melt. Simulation performed and figure created with with geodynamics code Aspect 2.0 (aspect.geodynamics.org).


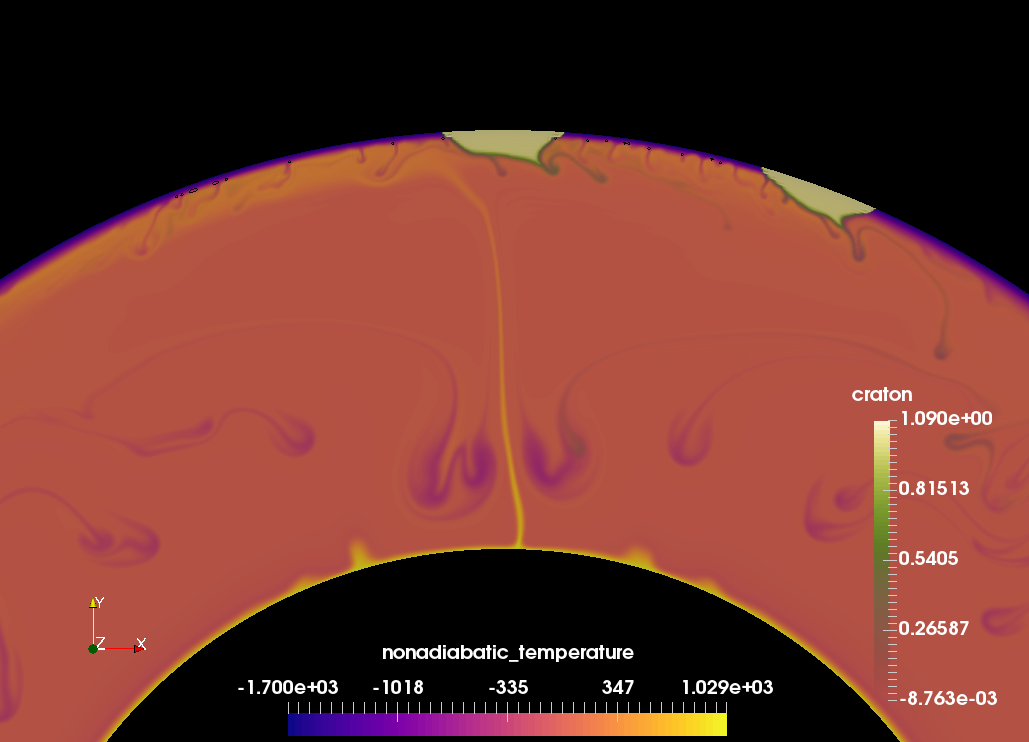


Supplementary Fig. 5 As above, black isocontours are for the “reduced” mantle solidus of Foley^51^, constrained to within the lithosphere. Note scale is zoomed in this Figure. Simulation performed and figure created with with geodynamics code Aspect 2.0 (aspect.geodynamics.org).


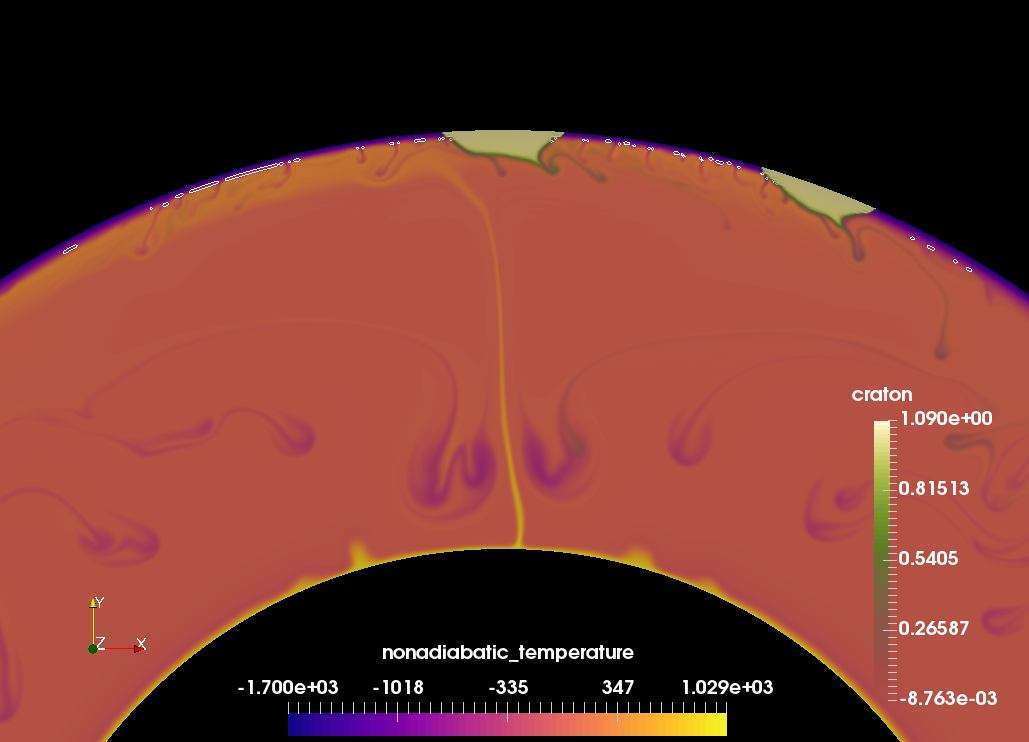


Supplementary Fig. 6 As above, the white isocontours are for an “oxidised” solidus of Foley^51^, constrained to within the lithosphere. Simulation performed and figure created with with geodynamics code Aspect 2.0 (aspect.geodynamics.org).

***Sensitivity to continental configuration***

We have tested the sensitivity of our results to continental configuration with the following models. One, possibly permitted by paleomagnetics, is that an ocean basin may have existed between the Kaapvaal and Pilbara cratons at ~2.05Ga. We test the implications of that here. Conversely, Supplementary Fig. 7 shows our preferred reconstruction model, which includes the data compilation in Figure 3 in the main text. Here we have a strong initial plume (d~800km) in combination with a lithospheric configuration that shows adjacent Kaapvaal and Pilbara cratons. In this case, material is advected laterally extremely efficiently, covering ~2000km in less than 1Myr.


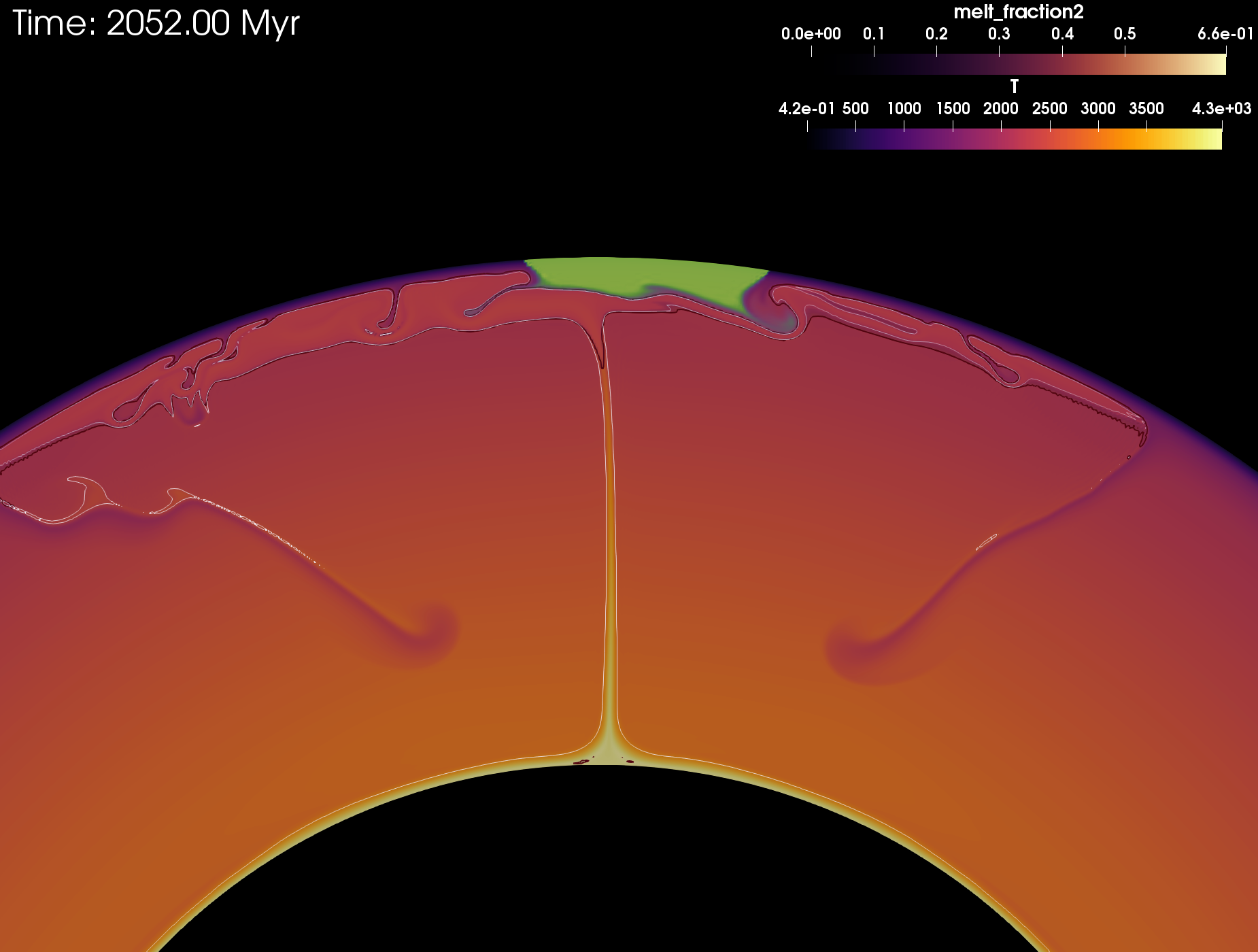


Supplementary Fig. 7 Lateral extent of plume material 3Myr after arrival of the primary plume beneath the Kaapvaal Craton. Initial plume head diameter is 800km in the lower mantle. Simulation performed and figure created with with geodynamics code Aspect 2.0 (aspect.geodynamics.org).

In the second example (Supplementary Fig. 8), the 2nd (Pilbara) Craton is located 2200km from the Kaapvaal impact point of the Bushveld plume. The contour is the 10% melt contour assuming no extraction, and the plume material eventually makes it past the Pilbara Craton keel. This was one of the smaller plumes (d<200km) tested, which took ~12Myr to cross the Pilbara Craton and reach the distal EGS region of the Yilgarn Craton ‘, which is outside the time constraints. Larger plumes cross this distance much faster.

Thus, the ultimate continental positioning will affect the timing of the arrival of the magmatic front, as well as exert a small control on whether small volume 'weak' plumes may advect around cratonic keels. However, over the range of possible Bushveld plume sizes, the exact continental configuration does not alter the fundamental conclusions, but may modify the size vs time relationship for plumes.


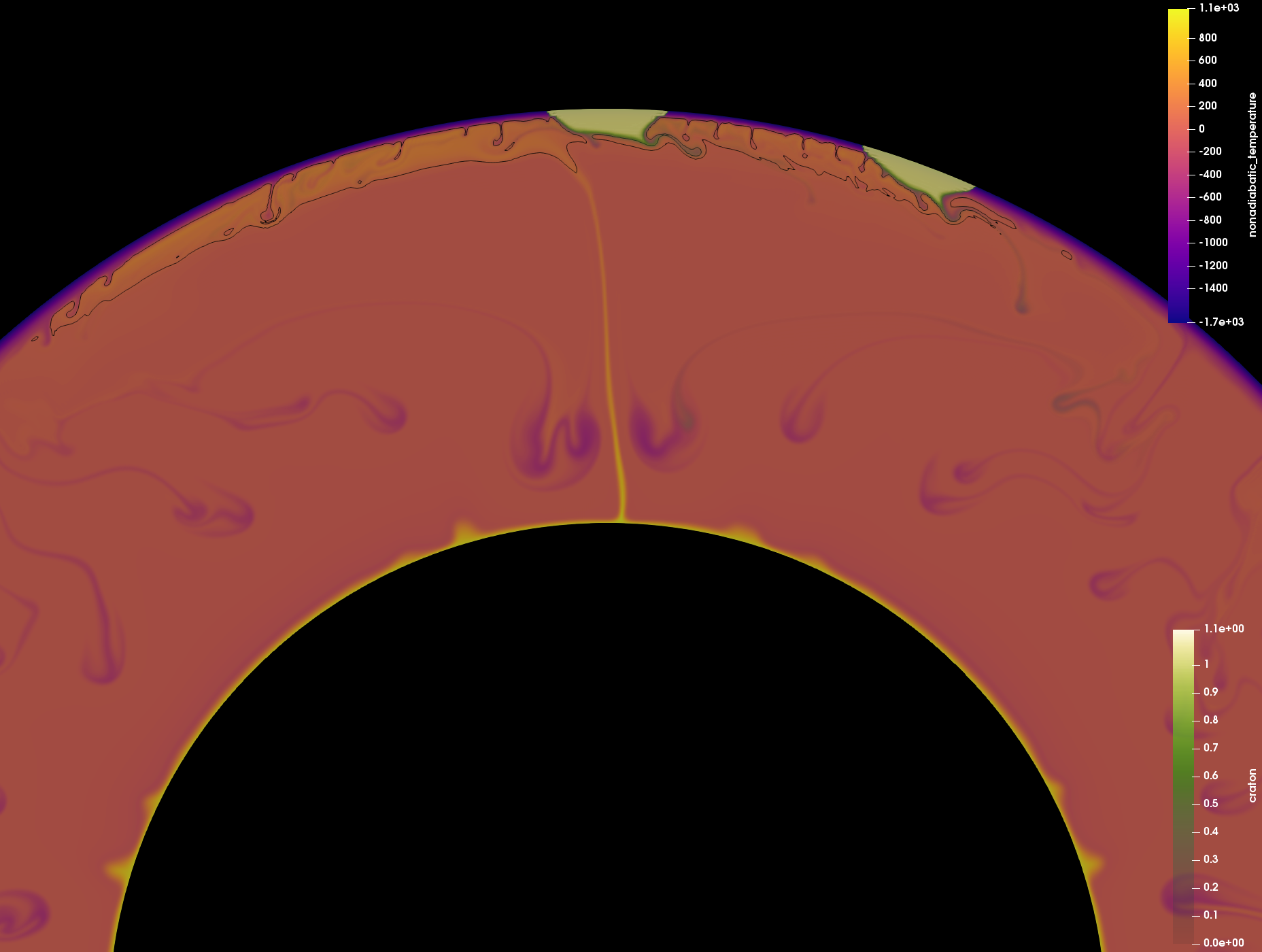


Supplementary Fig. 8 Here the Kaapvaal (middle) and Pilbara (right) cratons are separated by 2200km. The plume was weak (d<120km in the lower mantle), and the snapshot is for 12Myr after the arrival of the plume under the Kaapvaal Craton. However, the plume front was eventually able to traverse the Pilbara Craton keel into the distal EGS of the Yilgarn Craton. Simulation performed and figure created with with geodynamics code Aspect 2.0 (aspect.geodynamics.org).

**References**

1. Cassidy, K.F., Champion, D.C., Krapez, B., Barley, M.E., Brown, S.J.A., Bleweet, R.S., Groenewald, P.B., Tyler, I.M. A revised geological framework for the Yilgarn Craton, Western Australia. *Geological Survey of Western Australia* (2006).
2. Swager, C., Goleby, B., Drummond, B., Rattenbury, M., Williams, P. Crustal structure of granite-greenstone terranes in the Eastern Goldfields, Yilgarn Craton, as revealed by seismic reflection profiling. *Precambrian Research* **83**, 43-56 (1997).
3. Barley, M., Brown, S., Cas, R., Cassidy, K., Champion, D., Gardoll, S., Krapez, B. An integrated geological and metallogenic framework for the eastern Yilgarn Craton: developing geodynamic models of highly mineralised Archaean granite–greenstone terranes. *AMIRA Project P 624, Final Report* (2003).
4. Barley, M., Brown, S., Krapez, B., Cas, R. Tectonostratigraphic analysis of the eastern Yilgarn craton: An improved geological framework for exploration in Archaean terranes. *AMIRA Project P437A, Final Report* (2002).
5. Pawley, M., Romano, S., Hall, C., Wyche, S., Wingate, M. The Yamarna Shear Zone: a new terrane boundary in the northeastern Yilgarn Craton. Geological Survey of Western Australia, *Annual Review*, 26-32 (2007).
6. Pawley, M.J., Wingate, M.T.D., Kirkland, C.L., Wyche, S., Hall, C.E., Romano, S.S., Doublier, M.P. Adding pieces to the puzzle: episodic crustal growth and a new terrane in the northeast Yilgarn Craton, Western Australia. *Australian Journal of Earth Sciences* **59**, 603-623 (2012).
7. Czarnota, K., Champion, D., Goscombe, B., Blewett, R., Cassidy, K., Henson, P., Groenewald, P. Geodynamics of the eastern Yilgarn Craton. *Precambrian Research* **183**, 175-202 (2010).
8. Kent, A.J., Cassidy, K.F., Mark Fanning, C. Archean gold mineralization synchronous with the final stages of cratonization, Yilgarn Craton, Western Australia. *Geology* **24**, 879-882 (1996).
9. Mole, D.R., Fiorentini, M.L., Thebaud, N., Cassidy, K.F., McCuaig, T.C., Kirkland, C.L., Romano, S.S., Doublier, M.P., Belousova, E.A., Barnes, S.J., Miller, J. Archean komatiite volcanism controlled by the evolution of early continents. *Proceedings of the National Academy of Sciences* **111**, 10083-10088 (2014).
10. Graham, S., Lambert, D., Shee, S. The petrogenesis of carbonatite, melnoite and kimberlite from the Eastern Goldfields Province, Yilgarn Craton. *Lithos* **76**, 519-533 (2004).
11. Nemchin, A., Pidgeon, R., Wilde, S. Timing of Late Archaean granulite facies metamorphism in the southwestern Yilgarn Craton of Western Australia: evidence from U-Pb ages of zircons from mafic granulites. *Precambrian Research* **68**, 307-321 (1994).
12. Pisarevsky, S.A., De Waele, B., Jones, S., Söderlund, U., Ernst, R.E. Paleomagnetism and U–Pb age of the 2.4 Ga Erayinia mafic dykes in the south-western Yilgarn, Western Australia: Paleogeographic and geodynamic implications. *Precambrian Research* **259**, 222-231 (2015).
13. Smirnov, A.V., Evans, D.A., Ernst, R.E., Söderlund, U., Li, Z.-X. Trading partners: Tectonic ancestry of southern Africa and western Australia, in Archean supercratons Vaalbara and Zimgarn. *Precambrian Research* **224**, 11-22 (2013).
14. Wingate, M.T., Pirajno, F., Morris, P.A. Warakurna large igneous province: A new Mesoproterozoic large igneous province in west-central Australia. *Geology* **32**, 105-108 (2004).
15. Blewett, R.S., Henson, P.A., Roy, I.G., Champion, D.C., Cassidy, K.F. Scale-integrated architecture of a world-class gold mineral system: the Archaean eastern Yilgarn Craton, Western Australia. *Precambrian Research* **183**, 230-250 (2010).
16. Johnson, S.P., Thorne, A., Tyler, I., Korsch, R., Kennett, B., Cutten, H., Goodwin, J., Blay, O., Blewett, R., Joly, A. Crustal architecture of the Capricorn Orogen, Western Australia and associated metallogeny. *Australian Journal of Earth Sciences* **60**, 681-705 (2013).
17. Mole, D.R., Fiorentini, M.L., Thebaud, N., McCuaig, T.C., Cassidy, K.F., Kirkland, C.L., Wingate, M.T.D., Romano, S.S., Doublier, M.P., Belousova, E.A. Spatio-temporal constraints on lithospheric development in the southwest–central Yilgarn Craton, Western Australia. *Australian Journal of Earth Sciences* **59**, 625-656 (2012).
18. Wyche, S., Kirkland, C., Riganti, A., Pawley, M., Belousova, E., Wingate, M. Isotopic constraints on stratigraphy in the central and eastern Yilgarn Craton, Western Australia. *Australian Journal of Earth Sciences* **59**, 657-670 (2012).
19. Choi, E., Fiorentini, M., Giuliani, A., Foley, S., Maas, R., Taylor, W., Subduction-related petrogenesis of Late Archean calc-alkaline lamprophyres in the Yilgarn Craton (Western Australia). *Precambrian Research* **338**, 105550 (2020).
20. Hammerli, J., Kemp, A.I., Whitehouse, M.J. In situ trace element and Sm-Nd isotope analysis of accessory minerals in an Eoarchean tonalitic gneiss from Greenland: Implications for Hf and Nd isotope decoupling in Earth's ancient rocks. *Chemical Geology* **524**, 394-405 (2019).
21. Le Maitre, R., Streckeisen, A., Zanettin, B., Le Bas, M., Bonin, B., Bateman, P., Bellieni, G., Dudek, A., Efremova, S., Keller, J. Igneous rocks: A classification and glossary of terms; Recommendations of the International Union of Geological Sciences, Subcommission on the Systematics of Igneous rocks. *Cambridge University Press* (2002).
22. Pirajno, F., Gonzalez-Alvarez, I. Border, A., Porter, T. M. Mount Weld and Gifford Creek rare earth elements carbonatites. In; Australian ore deposits. The Australian Institute of Mining and Metallurgy, Melbourne, Monograph 32, Phillips G. N. (ed), pp 163-166 (2017).
23. Chakhmouradian, A.R., Reguir, E.P., Zaitsev, A.N., Couëslan, C., Xu, C., Kynický, J., Mumin, A.H., Yang, P. Apatite in carbonatitic rocks: Compositional variation, zoning, element partitioning and petrogenetic significance. *Lithos* **274**, 188-213 (2017).
24. Mitchell, R.H. Kimberlites: mineralogy, geochemistry, and petrology. Springer Science & Business Media (1986).
25. Tappe, S., Foley, S.F., Jenner, G.A., Kjarsgaard, B.A. Integrating ultramafic lamprophyres into the IUGS classification of igneous rocks: rationale and implications. *Journal of Petrology* **46**, 1893-1900 (2005).
26. Rock, N.M.S. Lamprophyres. Springer Science & Business Media (1991).
27. Tappe, S., Jenner, G.A., Foley, S.F., Heaman, L., Besserer, D., Kjarsgaard, B.A., Ryan, B. Torngat ultramafic lamprophyres and their relation to the North Atlantic Alkaline Province. *Lithos* **76**, 491-518 (2004).
28. Maas, R., Grew, E.S., Carson, C.J. Isotopic constraints (Pb, Rb-Sr, Sm-Nd) on the sources of early Cambrian pegmatites with boron and beryllium minerals in the Larsemann Hills, Prydz Bay, Antarctica. *The Canadian Mineralogist* **53**, 249-272 (2015).
29. Yaxley, G.M., Kamenetsky, V.S., Nichols, G.T., Maas, R., Belousova, E., Rosenthal, A., Norman, M. The discovery of kimberlites in Antarctica extends the vast Gondwanan Cretaceous province. *Nature communications* **4**, 2921 (2013).
30. Odin, G.S., 1982. Numerical dating in stratigraphy. *John Wiley & Sons*.
31. Williams, I., Tetley, N., Compston, W., McDougall, I., 1982. A comparison of K-Ar and Rb-Sr ages of rapidly cooled igneous rocks: two points in the Palaeozoic time scale re-evaluated. *Journal of the Geological Society* **139**, 557-568 (1982).
32. Thirlwall, M. Geochronology of Late Caledonian magmatism in northern Britain. *Journal of the Geological Society* **145**, 951-967 (1988).
33. Villa, I.M., De Bièvre, P., Holden, N., Renne, P. IUPAC-IUGS recommendation on the half life of 87Rb. *Geochimica et Cosmochimica Acta* **164**, 382-385 (2015).
34. Marillo-Sialer, E., Woodhead, J., Hergt, J., Greig, A., Guillong, M., Gleadow, A., Evans, N., Paton, C. The zircon ‘matrix effect’: evidence for an ablation rate control on the accuracy of U–Pb age determinations by LA-ICP-MS. *Journal of Analytical Atomic Spectrometry* **29**, 981-989 (2014).
35. Thompson, J., Meffre, S., Maas, R., Kamenetsky, V., Kamenetsky, M., Goemann, K., Ehrig, K., Danyushevsky, L. Matrix effects in Pb/U measurements during LA-ICP-MS analysis of the mineral apatite. *Journal of Analytical Atomic Spectrometry* **31**, 1206-1215 (2016).
36. Chew, D., Petrus, J., Kamber, B. U–Pb LA–ICPMS dating using accessory mineral standards with variable common Pb. *Chemical Geology* **363**, 185-199 (2014).
37. Paton, C., Woodhead, J.D., Hellstrom, J.C., Hergt, J.M., Greig, A., Maas, R. Improved laser ablation U‐Pb zircon geochronology through robust downhole fractionation correction. *Geochemistry, Geophysics, Geosystems* 11 (2010).
38. Ludwig, K.R. Isoplot 3.00: A geochronological toolkit for Microsoft Excel. *Berkeley Geochronology Center Special Publication* 4, 70 (2003).
39. Vermeesch, P., 2018. IsoplotR: a free and open toolbox for geochronology. *Geoscience Frontiers* **9**, 1479-1493 (2018).
40. Stacey, J.t., Kramers, J. Approximation of terrestrial lead isotope evolution by a two-stage model. *Earth and Planetary Science Letters* **26**, 207-221 (1975).
41. Nelson, D., Chivas, A., Chappell, B., McCulloch, M. Geochemical and isotopic systematics in carbonatites and implications for the evolution of ocean-island sources. *Geochimica et Cosmochimica Acta* **52**, 1-17 (1988).
42. Collerson, K.D. Geochemistry and Rb-sr geochronology of associated proterozoic peralkaline and subalkaline anorogenic granites from Labrador. *Contr. Mineral. and Petrol.* **81,**126–147 (1982).
43. Jaireth, S., Hoatson, D.M., Miezitis, Y. Geological setting and resources of the major rare-earth-element deposits in Australia. *Ore Geology Reviews* 62, 72-128 (2014).
44. White, B. The geochronology and thermochronology of the Brockman Creek 01, Melita 01 and Melita 02 kimberlites, Western Australia. *Unpublished Honours thesis University of Melbourne* (2000).
45. Kronbichler, M., Heister, T., Bangerth, W. High accuracy mantle convection simulation through modern numerical methods. *Geophysical Journal International* **191**, 12-29 (2012).
46. Stixrude, L., de Koker, N., Sun, N., Mookherjee, M., Karki, B.B. Thermodynamics of silicate liquids in the deep Earth. *Earth and Planetary Science Letters* **278**, 226-232 (2009).
47. Ringwood, A., Irifune, T. Nature of the 650–km seismic discontinuity: implications for mantle dynamics and differentiation. *Nature* **331**, 131 (1988).
48. Garel, F., Goes, S., Davies, D., Davies, J.H., Kramer, S.C., Wilson, C.R. Interaction of subducted slabs with the mantle transition‐zone: A regime diagram from 2‐D thermo‐mechanical models with a mobile trench and an overriding plate. *Geochemistry, Geophysics, Geosystems* **15**, 1739-1765 (2014).
49. O'Neill, C.O., Lenardic, A., Moresi, L., Torsvik, T.H., Lee, C.-T.A. Episodic Precambrian subduction. *Earth and Planetary Science Letters* **262**, 552-562 (2007).
50. Foley, S.F. Rejuvenation and erosion of the cratonic lithosphere. *Nature Geoscience* **1**, 503 (2008).
51. Vermeesch, P. On the visualisation of detrital age distributions. *Chemical Geology* **312-313**, 190-194 (2018).
